# A review of the botany, phytochemistry, traditional uses, pharmacology, toxicology, and quality control of the *Astragalus memeranaceus*

**DOI:** 10.3389/fphar.2023.1242318

**Published:** 2023-08-23

**Authors:** Panpan Wang, Zhen Wang, Zhanping Zhang, Huiyan Cao, Lingyang Kong, Wei Ma, Weichao Ren

**Affiliations:** School of Pharmacy, Heilongjiang University of Chinese Medicine, Harbin, China

**Keywords:** *Astragalus memeranaceus*, ethnopharmacology, botany, traditional uses, phytochemistry, pharmacology, toxicology

## Abstract

Astragali Radix (Huangqi) is mainly distributed in the Northern Hemisphere, South America, and Africa and rarely in North America and Oceania. It has long been used as an ethnomedicine in the Russian Federation, Mongolia, Korea, Kazakhstan, and China. It was first recorded in the Shennong Ben Cao Jing and includes the effects of reinforcing healthy qi, dispelling pathogenic factors, promoting diuresis, reducing swelling, activating blood circulation, and dredging collaterals. This review systematically summarizes the botanical characteristics, phytochemistry, traditional uses, pharmacology, and toxicology of Astrag*alus* to explore the potential of Huangqi and expand its applications. Data were obtained from databases such as PubMed, CNKI, Wan Fang Data, Baidu Scholar, and Google Scholar. The collected material also includes classic works of Chinese herbal medicine, Chinese Pharmacopoeia, Chinese Medicine Dictionary, and PhD and Master’s theses. The pharmacological effects of the isoflavone fraction in Huangqi have been studied extensively; The pharmacological effects of Huangqi isoflavone are mainly reflected in its anti-inflammatory, anti-tumor, anti-oxidant, anti-allergic, and anti-diabetic properties and its ability to treat several related diseases. Additionally, the medicinal uses, chemical composition, pharmacological activity, toxicology, and quality control of Huangqi require further elucidation. Here, we provide a comprehensive review of the botany, phytochemistry, traditional uses, pharmacology, toxicology, and quality control of Astragalus to assist future innovative research and to identify and develop new drugs involving Huangqi.

## Introduction

*Astragalus* L. is the largest genus in the family Leguminosae comprising approximately 2,900 species. *Astragalus membranaceus* (Fisch.) Bunge and *Astragalus membranaceus* (Fisch.) Bge. Var. *mongholicus* (Bge) Hsiao are used worldwide because of their high medicinal and nutritional value (Wu et al., 2018). Astragali Radix (Huangqi), the dried roots of *A. membranaceus* or *Astragalus mongholicus*, is commonly used as a herbal ethnopharmacological herb in China. Huangqi is mainly distributed in the Russian Federation, Mongolia, and China (Li et al., 2017a). The application of Huangqi can be traced back to the Han Dynasty and was first recorded in Shennong Ben Cao Jing (Han Dynasty, BCE 202–220), where it was categorized as a high-quality product. Li Shizhen’s “Compendium of the Materia Medica” (Ming Dynasty, AD 1552–1578) lists Huangqi as the first tonic herb, which mainly reinforced healthy qi, dispelling pathogenic factors, promoting diuresis, and reducing swelling. Huangqi has been prevalent for more than 2,000 years with over 200 types of herbal decoctions and has experienced extensive clinical application in Chinese medicine (Yuan et al., 2012).

The chemical composition of *Astragalus* is complex and mainly includes flavonoids, saponins, and polysaccharide compounds, as well as amino acids and trace elements (Wang et al., 2021a). To date, more than 200 compounds have been isolated from Astragalus species, among which isoflavones such as calycosin (CAL), calycosin-7-glucoside (CG), formononetin (FMN), and ononin (ON) have significant value because of their significant antioxidant, anticancer, anti-inflammatory, and neuroprotective pharmacological effects (Jin et al., 2014; Yu et al., 2018b). Modern pharmacological studies have verified that Huangqi has various pharmacological activities, which can improve the body’s immunity; scavenge free radicals; and exert anti-inflammatory, anti-tumor, anti-diabetic, and antioxidant effects (Wu et al., 2016). The aqueous extracts of Huangqi are often used separately or in combination with other drugs to expand the range of its medicinal effects. For example, the combined use of *Astragalus* and *Angelica* in Angelica blood tonic soup can improve the deficiency of both qi and blood (Ning et al., 2002). In addition, Huangqi is rich in Astragalus polysaccharides (Shi et al., 2014; Xue et al., 2015), which can treat severe acute respiratory syndrome coronavirus-2 (SARS-CoV2) infection. The combination of Huangqi and *Lonicera japonica* Thunb. has exhibited significant anti-SARS-CoV2 activity (Yeh et al., 2021). Safety evaluation studies on the toxicological effects of Huangqi have also received extensive attention. The main factors responsible for its pharmacological effects are closely related to its complex chemical composition and chemical-component interactions. Moreover, its wide range of biological activities makes Huangqi an extremely valuable medicinal resource.

As a rare botanical, Huangqi has attracted much attention because of its unique medicinal value and health effects. At present, the research on Huangqi mainly focuses on its chemical composition and pharmacological activity. However, there is a lack of comprehensive and up-to-date information about Huangqi. In this study, the literature on Huangqi since 1983 was collected, and the duplicated and irrelevant literature was removed. This review systematically summarized the literature on the botany, phytochemistry, traditional uses, pharmacology, toxicology, and quality control of Huangqi. This review aims to comprehensively and objectively understand Huangqi, solve the problems in its application, explore its inherent potential, and provide new ideas for future innovative research and the search for new drugs.

### Botany

**FIGURE 1:**
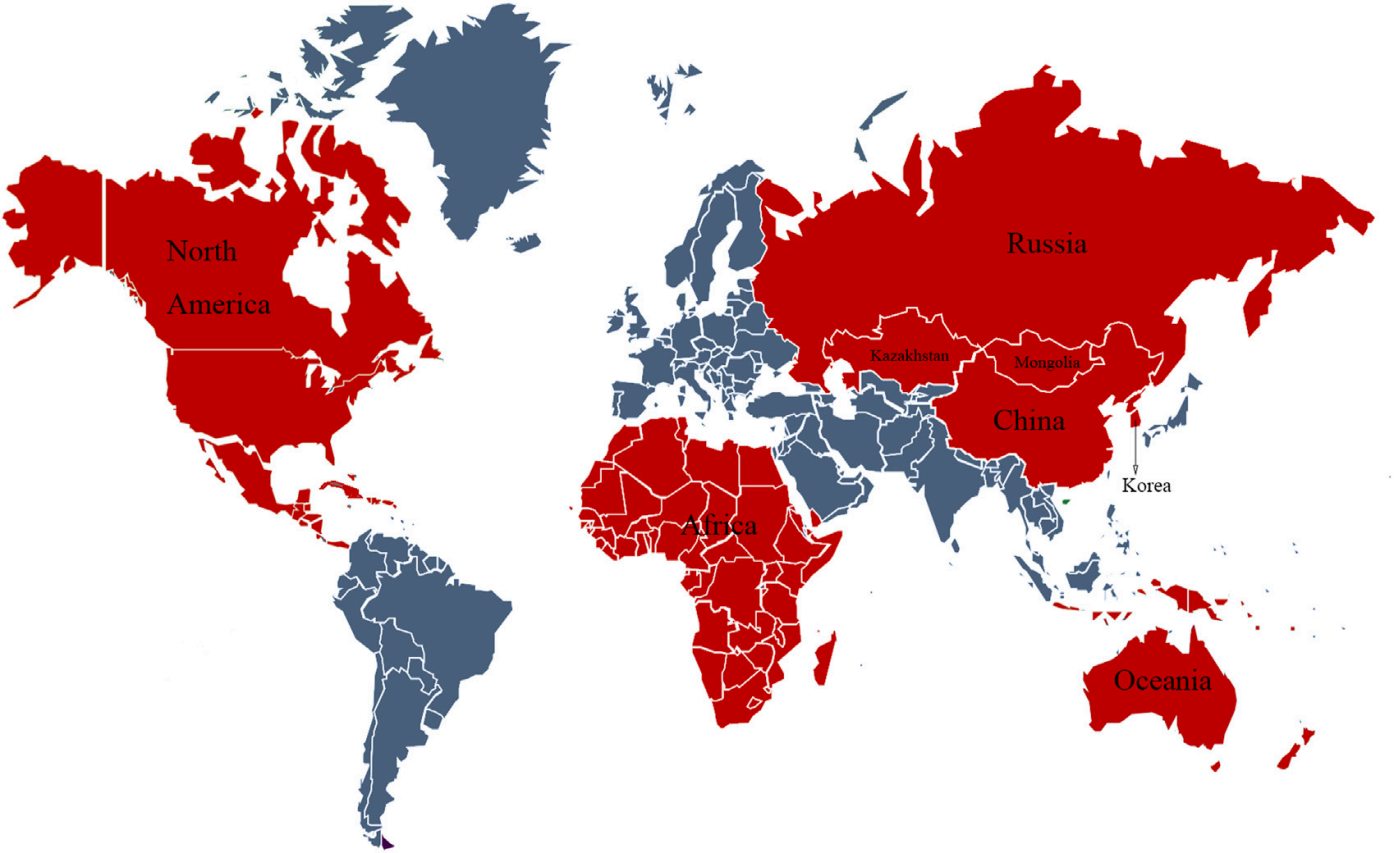
World map of Huangqi distribution.

**FIGURE 2:**
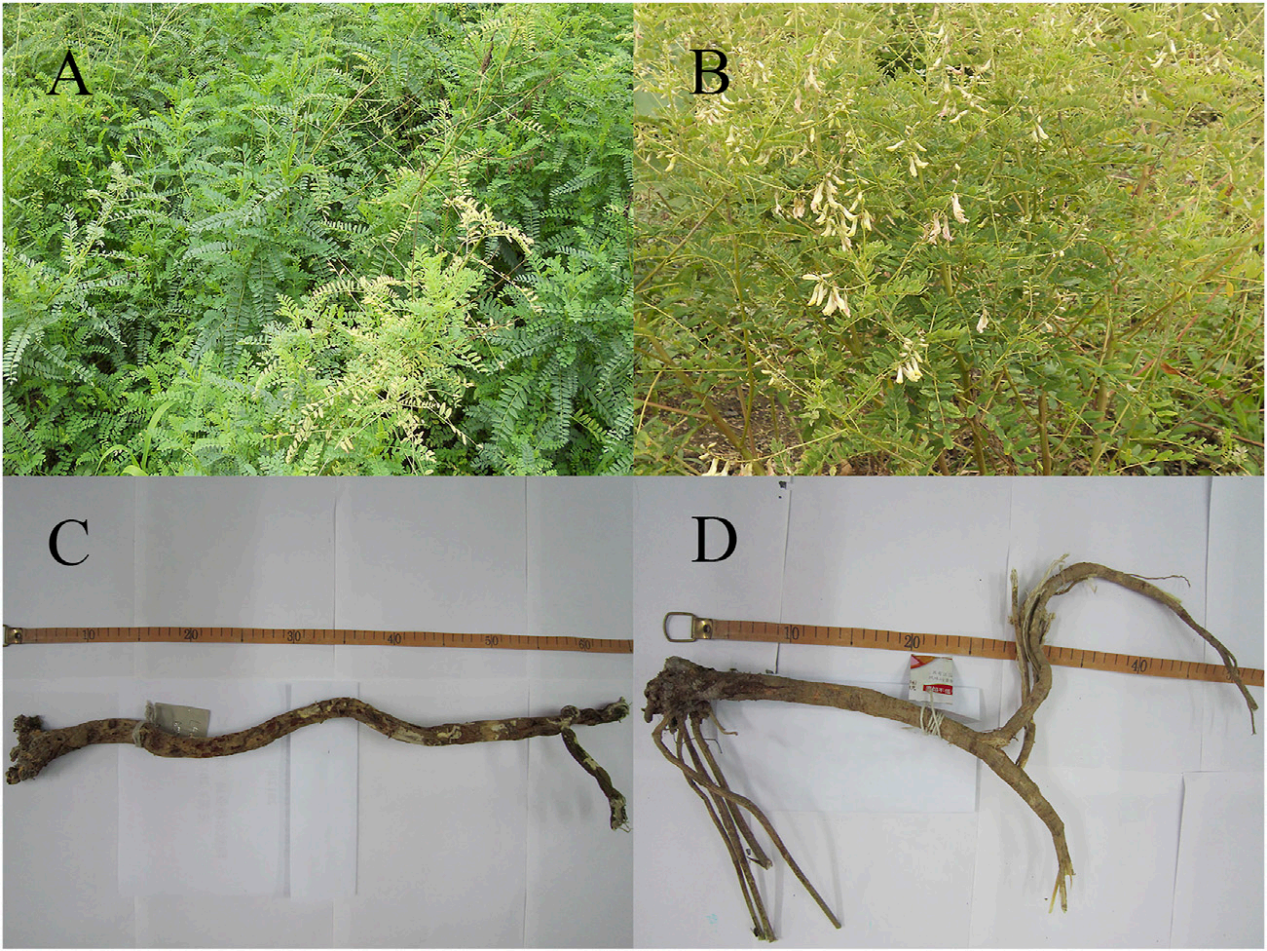
**(A)** *A. mongholicu* above ground parts; **(B)** *A. membranaceus* above ground parts; **(C)** *A. mongholicus* dry roots; **(D)** *A. membranaceus* dry roots.

*Astragalus* is widely distributed in the Northern Hemisphere, South America, and Africa; is rare in North America and Oceania; and is used as an ethnomedicine in the Russian Federation, Korea, Mongolia, Kazakhstan, and China (Figure 1). *Astragalus membranaceus* has the following morphological features: stems 60–150 cm tall, villous; leaves pinnately compound; leaflets 21–31 mm, ovate-lanceolate or elliptic, 7–30 mm long, 4–10 mm wide, white villous on both surfaces; leaf rachis villous; stipules narrowly lanceolate, 4–6 mm white villous; racemes axillary; flowers with striated bracts below; ovary hairy with an ovary stalk; pods membranous, swollen, ovate-tortuous, long-stalked, black pubescent (Figures 2B, D).

### Red indicates the use and distribution areas of Huang Q in the world, and blue indicates no distribution

*Astragalus mongholicus* is smaller than the original variety, with smaller leaflets (5–10 mm long and 3–5 mm wide) and glabrous pods and grows in an environment similar to that of *A. membranaceus*, such as sunny grasslands, thickets, and mountain slopes. The roots of these species can be used as a medicine and make a strong tonic that nourishes the kidneys, tonifies the spleen, prevents sweating, expels excess water, and eliminates swelling and pus. *A. mongholicus* grows on forest edges, thickets, sparse forests, meadows, and mountain slopes and is one of the most commonly used Chinese herbs (Figure 2A, C). *Astragalus* flowers in June–August and produces fruits in July–September. Good quality *Astragalus* plants are harvested at 4–5 years of age; transplanted seedlings can be harvested after 3 years. Plants can be harvested in autumn (August–September), after the branches and leaves wither, or in spring (March–April), before the plant sprouts. Plants are dug out after removing the soil and the stems, seedlings, and roots are cut off and dried in the Sun until they have dried by 60% or 70%. Then, they are arranged into small bundles stacked up together, natural pan sugar is added, sundried until soft, rubbed by hand, and then sundried completely (Sun, 2015). Research has revealed that the best harvesting period for *Astragalus* is from late October to mid-November when it has the highest yield, the best traits, and the best quality. The best time to harvest Huangqi is on a sunny day, and the entire root should be dug deeply to prevent the quality from being reduced through the breakage of the main root. The cleaning method of Huangqi greatly influences the content of the active ingredients, such as astragaloside IV and GC (Tang, 2022).

## Phytochemistry

To date, more than 200 compounds have been isolated from *Astragalus* species, including flavonoids, triterpenoids, polysaccharides, amino acids, alkaloids, *ß*-sitosterol, metalloids, and anthraquinones Supplementary Tables S1, S2. Among these, flavonoids and triterpenoids are the most abundant and polysaccharides, isoflavonoids, and triterpenoid saponins are the main active compounds of Huangqi responsible for its various pharmacological properties; these chemical components have been extensively studied (Song et al., 2007).

### Flavonoids

More than 100 flavonoid compounds have been isolated from *A. membranaceus*, with isoflavones, flavonoids, isoflavanes, and pterocarpans as the four major groups (Zhang et al., 2021a). Based on their structures, flavonoids can be divided into flavonols, flavones, flavanones, flavanols, anthocyanins, isoflavones, dihydro flavonols, and chalcones. Isoflavones are the most abundant among them, accounting for 80% of the total flavonoid content and are the signature Huangqi flavonoid compound (Huang et al., 2009). FMN and CAL, two important isoflavones in Huangqi, have been widely studied for their multiple pharmacological functions (Gao et al., 2014). CG has been demonstrated to possess various pharmacological activities, including antioxidant, anti-inflammatory, and neuroprotective activities (Choi et al., 2007; Jian et al., 2015). More importantly, CG has been described as a chemical indicator for the quality control of Huangqi in the Chinese Pharmacopoeia (2020).

### Saponins

*Astragalus* is rich in saponins, with triterpene saponins being the unique bioactive compounds. Saponins such as astragaloside I-VIII, isoastragaloside I-II, acetyl astragaloside, and soy saponin l and more than 100 triterpenoids have been isolated from *Astragalus* (Kitagawa et al., 1983a; Kitagawa et al., 1983b; Wang et al., 1983; Guo et al., 2019). Further, several cyclohexane-type tetracyclic triterpenes and oleanolane-type pentacyclic triterpenes, which are triterpene glycosides that contain a 30 carbon-atom skeleton, have been isolated from *Astragalus*. Astragalosides I, II, and IV are the most abundant saponins isolated from *Astragalus* roots. Astragaloside IV, which has significant pharmacological activities, has been studied extensively and described as one of the important indicators for the quality control of Huangqi in the Chinese Pharmacopoeia (Choi et al., 2007; Qi et al., 2008).

### Polysaccharides

More than 30 types of Astragalus polysaccharide, one of the main components in Huangqi, have been isolated from *Astragalus*, which are mainly divided into dextrans and heteropolysaccharides (Guo et al., 2019). Additionally, rhamnose, xylose, glucose, galactose, mannose, and alcohol-soluble polysaccharide (ASP) have been isolated from *Astragalus*. Alcohol-soluble polysaccharide is a neutral polysaccharide composed of mannose, glucose, galactose, and arabinose with pyranose rings and α-glycosidic bonds (Yu et al., 2018a). Recently, a new soluble sugar named APS4 has been isolated from *Astragalus*. The average molecular weight of APS4, composed of rhamnose, arabinose, xylose, mannose, and galactose, is approximately 1.5 × 10^3^ kDa, as revealed using high-performance gel permeation chromatography. APS4 has been demonstrated to have potential applications in cancer therapy (Yu et al., 2019).

### Others

*Astragalus* species also contain molybdenum, copper, manganese, scandium, rubidium, selenium, chromium, cobalt, cesium, iron, zinc, and more than 20 trace elements; however, the iron, manganese, zinc, and aluminum contents are higher than those of the others (Fu et al., 2014). In addition, *Astragalus* species contains 20 amino acids, including arginine, aspartic acid, asparagine, proline, and alanine (Shao et al., 2004). Further, other compounds such as folic acid, palmitic acid, bitter elements, coumarin, chloric acid, coumaric acid, choline, linolenic acid, legume sterols, ferulic acid, isoferulic acid, hydroxyphenyl acrylic acid, deerskinol, betaine, caffeic acid, linoleic acid, and β-sitosterol have also been identified from *Astragalus* species.

### Traditional uses

Huangqi is named so because of its yellow color and significant tonic potential. Huangqi contains various active ingredients and thus has a wide range of pharmacological effects, playing an important role in traditional Chinese medicine. Huangqi supplements the qi, solidifies the surface, benefits water, supports toxins, and generates muscles. As a traditional Chinese medicine, it is mainly used for treating spleen and stomach weakness, qi deficiency and blood withdrawal, qi deficiency and edema, chronic nephritis, ulcers, or ulcers that remain uncured. In China, it is known as the “Little Ginseng of Northeast China” (Napolitano et al., 2013). The Dictionary of Traditional Chinese Medicine records that Huangqi is taken in a decoction of 9–15 g. Large doses include 30–60 g (Li, 2005). It is suitable for stir-baking with an adjuvant to tonify and replenish the middle qi and used raw to secure the exterior, induce diuresis, and expel toxins. Huangqi has been used clinically in a variety of classical prescriptions. Related formulations of Huangqi with other herbs are shown in Table 1.

**TABLE 1:** Traditional and clinical uses of Huangqi in China.

NO.	Prescription name	Major component	Formulation	Traditional and clinical uses	Ref
1	Bu-zhong-yi-qi- tang	Huangqi, Renshen, Zhigancao, Baizhu, Chenpi, Shenma, Chaihu	Decoction	Chronic gastritis, gastroptosis, myasthenia gravis, uterine prolapse	Treatise on the Spleen and Stomach《脾胃论》
2	Yiqi congming tang	Huangqi, Renshen, Gancao, Shenma, Gegen, Manjingzi, Baishao, Huangbai	Decoction	Vertigo, cervical spondylosis, cerebral arteriosclerosis, hypertension, tinnitus, dementia	Treatise on the Spleen and Stomach《脾胃论》
3	Shengxian tang	Huangqi, Zhimu, Chaihu, Shenma, Gegen	Decoction	Sick sinus syndrome, chronic fatigue syndrome, gastroptosis, coronary heart disease, diarrhea	A Record of Medieval References to the West in Medicine《医学衷中参西录》
4	Danggui buxue tang	Huangqi, Danggui	Decoction	Leukopenia, functional uterine bleeding, diabetic nephropathy, nephrotic syndrome	Treatise on the Confusion of Internal and External Injuries《内外伤辨惑论》
5	Guipi tang	Huangqi, Renshen, Baizhu, Zhigancao, Fuling, Danggui,Longyanrou, Suanzaoren, Yuanzhi, Muxiang	Decoction	Insomnia, palpitations, depression, facial spasm	Ji Sheng Fang《济生方》
6	Danggui liuhuang tang	Huangqi, Shengdihuang, Shudihuang, Huangqin, Huangbai, Huanglian	Decoction	Night sweats, perimenopausal syndrome, hyperthyroidism, chronic prostatitis, viral myocarditis	Secret Record of the Chamber of Orchids《兰室秘藏》
7	Huangqi guizhi wuwu tang	Huangqi, Baishao, Guizhi, Shengjiang, Dazao	Decoction	Diabetes mellitus, cervical spondylosis	The Golden Chamber《金匮要略》
8	Huangqi jianzhong tang	Huangqi, Shaoyao, Guizhi, Zhigancao, Shengjiang, Dazao, Maltose	Decoction	Reflux esophagitis, chronic atrophic gastritis, gastric ulcer, stable chronic obstructive pulmonary disease, gastric cancer	The Golden Chamber《金匮要略》
9	Buyang huanwu tang	Huangqi, Danggui, Chishao, Dilong, Chuanxiong, Taoren, Honghua	Decoction	Hemiplegia, mouth and eye deviation, frequent urination	Yilin Correcting Mistakes《医林改错》
10	Fangji Huangqi tang	Fangji, Huangqi, Baizhu, Gancao, Shengjiang, Dazao	Decoction	Rheumatism	The Golden Chamber《金匮要略》
11	Taishan panshi san	ZhiHuangqi, Renshen, Danggui, Chuangxiong, Baishao, Dihuang, Baizhu, Duanxu, Sharen, Huangqin, Zhigancao, Glutinous rice	Powder	Shortness of breath and fatigue, palpitations and insomnia, dizziness in the head, and pale tongue	Jingyue’s Complete Works 《景岳全书》
12	Shengyu tang	Huangqi, Dangshen, Danggui, Chuanxiong, Baishao, Shudihuang	Decoction	Menorrhagia, oligomenorrhea, bleeding, prolonged menstrual period, infertility, coronary heart disease, angina pectoris, cervical spondylosis	Secret Record of the Chamber of Orchids《兰室秘藏》
13	Neibu Huangqi tang	Huangqi, Renshen, Fuling, Danggui, Chuanxiong, Baishao, Shudihuang, Rougui, Maidong, Yuanzhi, Gancao	Decoction	Non-infectious fever, duodenal ulcer, chronic suppurative osteomyelitis, diabetes mellitus after orthopedic surgery	Surgical Masamune《外科正宗》
14	Yupingfeng san	Huangqi, Baizhu, Fangfeng	Pill	Cold, night sweats, allergic rhinitis	Effective Formulae Handed Down for Generations《世医得效方》
15	Duli san	Calcinated oyster, Huangqi, Mahuang root	Powder	Spontaneous sweating and night sweating caused by postpartum, postoperative, pulmonary tuberculosis, and autonomic dysfunction	Prescription of Peaceful Benevolent Dispensary《太平惠民和剂局方》

Huangqi has a long history and is widely used in classical formulations. Huangqi was first recorded as a high grade herb in the Shennong Ben Cao Jing 《神农本草经》 (Han Dynasty, BCE 202–220), and Li Shizhen’s Compendium of Materia Medica 《本草纲目》 (Ming Dynasty, AD 1552–1578) lists Huangqi as the leading tonic medicine. In Zhang Zhongjing’s the Golden Chamber 《金匮要略》 (Eastern Han Dynasty, AD 200–210), the dosage and preparation of different medicinal formulations are described in detail, and Huangqi is mentioned in eight of these formulations: Huangqi Gui Zhi Wuwu Tang, Huangqi Jianzhong Tang, Fangji Huangqi Tang, Fangji Gui Zhi Tang, Wutou Tang, Gui Zhi Tang, Huangqi Peony Bitter Wine Tang, and Qianjin Sanhuang Tang.However, there are no records of prescribing Huangqi for febrile diseases 《伤寒论》 (Dong Han Dynasty, AD 25–220). In the Secret Record of the Chamber of Orchids 《兰室秘藏》 (Yuan Dynasty, AD 1115–1368), written by Li Dong Yuan, Huangqi is mentioned 19 times as a tonifying agent of the spleen (Liu et al., 2022). In Nei Wai Shang Bian Huo Lun 《内外伤辩惑论》 (Yuan Dynasty, AD 1232–1247), Huangqi is mentioned 11 times in grouping frequency and nine times in combination with ginseng for its effectiveness in benefiting the qi and strengthening the spleen. Huangqi is mentioned 15 times in the Treatise on the Spleen and Stomach 《脾胃论》 (Yuan Dynasty, AD 1249) as an agent widely used to tonify the deficiencies of the spleen and stomach. Wu Jutong wrote Warm Disease Argument 《温病条辩》 (Qing Dynasty, A.D. 1644–1911), where in addition to borrowing Qingshu Yiqi Tang and Buzhong Yiqi Tang and formulating his own formula, he only used the addition and subtraction of Buzhong Yiqi Tang to treat “Qi deficiency and lower trapping, the portal does not hide.” According to Wang Qingren, Huangqi is the preferred qi tonic for treating Yuan Qi deficiency. In 11 of the 33 prescriptions in the Medical Forest Correction 《医林改错》 (Qing Dynasty, AD 1830), Huangqi has been mentioned as the most abundant agent (Sha, 2014). Jing Yue advocated warm tonicity and proposed the idea of “Yang Fei You Yu.” He used qi tonicity in several formulas, including Huangqi in 42 of the 132 formulas (Yi, 2013). In the Orthodox Manual of External Medicine 《外科正宗》 (Ming Dynasty, AD 1617), Huangqi appears 10 times among 32 main treatment formulas for swollen ulcers. Chen Yuren believed that swollen ulcers were caused by “weakness of the spleen and stomach and weakened Yang Qi,” and Huangqi was used in the formulas to nourish qi deficiency and strengthen the spleen (Zhao et al., 2018). Huangqi appears 25 times as a tonic for deficiency, thirst, sores, and fractures in the Prescription of Peaceful Benevolent Dispensary 《太平惠民和剂局方》 (Ming Dynasty, A.D. 1078–1085), written by the Song Dynasty Hodong Bureau (Wang, 2021).

In addition to the studies on Huangqi in classical medicine, it has been extensively studied recently. In Records of Chinese Medicine with Reference to Western Medicine《医学衷中参西录》, Zhang Xichun mentioned Huangqi 35 times for its tonic power that promotes myogenesis, solid qi, diuresis and prevents the collapse of the belt. He also created four formulas of Shengxian Tang, all with Huangqi as the leading substance, that tonify and increase the qi and treat qi trapped in the chest. Zhang Xichun believed that some drugs need to be used raw to obtain the complete benefits of the medicine, and heating weakens concoctions, making them ineffective or may even cause them to have the opposite effect. Thus, we believe that with the increased understanding of the pharmacology and pharmacological effects of Huangqi, its therapeutic effects have also enriched and improved.

### Pharmacology

**FIGURE 3:**
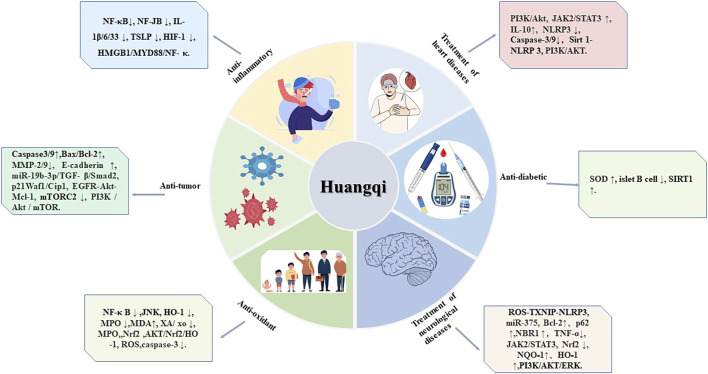
Six pharmacological effects of Huangqi. This figure shows the most highlighted six effects in studies on Huangqi.

The medicinal component of *Astragalus* is its dried root. Modern pharmacological studies have shown that Huangqi has a wide range of immunological activities and is widely used as an immunostimulant, antioxidant, hepatoprotectant, diuretic, and expectorant. In recent years, astragalus isoflavones have been widely used because of their anti-inflammatory, anti-tumor, treatment of heart diseases, treatment of neurological diseases, anti-diabetic, and Anti-oxidant effects This review discusses the pharmacological effects of the isoflavone compounds in *Astragalus* Figure 3 and Table 2 to assist further scientific research.

**TABLE 2:** The pharmacological effects of Astragalus isoflavones.

Pharmacological effects	Extracts/Compounds	Types	Animal/cell	Dosage	Effects		Ref
anti-inflammatory action	Claycosin, Formononetin	*In vivo*	BALB/c mice	FMN (5 mg/kg) CAL (0.5, 5, 10 mg/kg)	Inhibition of TSLP production through regulation of NF-kB activation and translocation		Shen et al. (2014)
	Formononetin	*In vivo*	BALB/c mice	0,2,4,10 mg/kg	Decreased TSLP/IL-33 levels and increased E-calcineurin		Li et al. (2018)
	Claycosin	*In vitro*	Chondrocyte	1, 5, 10, 20 μg/mL	Blocking IL-1 by inhibiting the PI3K/AKT/FOXO1 pathway *ß* Induced chondrocyte apoptosis, inflammation, and ECM degradation		Guo et al. (2022)
	Claycosin	*In vivo*	C57BL/6J mice	5, 10, 20 mg/kg	Organization of κB-α, increased protein expression of p κB-α and NF-Bκ. The expression of B and p65 proteins is inhibited		Yujun et al. (2022)
	Formononetin	*In vivo*	SD mice	25, 50, 100 mg/kg	Inhibiting the NF-JB pathway reduces inflammation and promotes gastric mucosal angiogenesis		Yi et al. (2022)
	calycosin	*In vivo*	BALB/c mice	2, 10, 50 mg/kg	Adjusting HIF-1 in AD α, used as a potential anti allergic and barrier repair agent. Significantly reduce HIF-1 α Express and repair TJs		Jia et al. (2018)
	Formononetin	*In vitro*	BALB/c mice/HaCaT cell	10 mg/kg	Significantly promotes the expression of A20 protein and its mRNA, while suppressing the expression of TSLP protein and its mRNA.		Yuan et al. (2021)
	Formononetin	*In vivo*	Chondrocytes	10 mg/kg	Inhibiting the production of inflammatory mediators and cytokines, inhibiting the expression of cyclooxygenase-2 and inducing nitric oxide synthase, inhibiting the synthesis of metabolic factors such as MMPs and thrombospondin-5		Jia et al. (2022)
	Calycosin	*In vivo*/*in vitro*	SD rats	7.5, 15, 30 μg/mL	Inhibited inflammation and oxidative stress, improved damage to AEC-II cells, LPS or CLP induced HMGB1/MYD88/NF- κ; activation of B pathway and NLRP inflammatory tissue		Chen et al. (2021)
anti-tumor effect	Formononetin	*In vitro*	A2780 cell	0, 80, 160, 240 μg/mL	The ratio of Caspase3/9 and Bax/Bcl-2 protein expression was increased; reduced MMP-2/9 protein expression and ERK phosphorylation levels		Zhang et al. (2018)
	Formononetin	*In vitro*	SK-OV3 cell	35, 70, 140 μmol/L	Increasing E-cadherin and decreasing MMP-9 expression to inhibit migration and invasion of ovarian cancer SKOV-3 cells		Gu et al. (2020)
	Formononetin	*In vitro*	SK-OV3 cell	35, 70, 140 μmol/L	Acting on miR-19b-3p/TGF- β/The Smad2 signaling pathway inhibits the proliferation, migration, and invasion of SKOV3 in ovarian cancer cells, inducing their apoptosis		Niu et al. (2021)
	Calycosin	*In vitro*	PDACcell	50 μg/mL	Inhibition of cell growth by inducing cell cycle arrest and caspase-dependent apoptosis induced by p21Waf1/Cip1		Zhang et al. (2020)
	Formononetin	*In vivo*/*in vitro*	HeLa, SK-Hep1, HCT116 HuVECs cell	0, 10, 30, 50, 100 μg/mL	Inhibit PD-L1 cell proliferation and cell migration and promote apoptosis of tumor cells		Wang et al. (2022)
	Formononetin	*In vivo*/*in vitro*	NSCLC cell	0.5, 1.5, 3, 10 μg/mL	Inhibition of EGFR-Akt-Mcl-1 axis to suppress tumor growth in non-small cell lung cancer		Yu et al. (2020)
	Formononetin	*In vitro*	MDA-MB-468 cell	50 mg/kg	Inhibition of mTORC2 enhances the inhibitory effect of everolimus on the mTOR pathway		Zhou et al. (2019)
	campanulin, ononin, calycosin formononetin	*In vitro*	MCF-7, SK-BR-3, MDA-MB-231 cell	25, 50, 100 μg/mL	Inhibition of cell proliferation and induction of apoptosis via PI3K/Akt/mTOR pathway		Zhou et al. (2018)
	Calycosin	*In vitro*	A549, H1299, BEAS-2B cell	25, 50, 100 μg/mL	Inhibition of LUAD cell colony formation, invasion, migration and EMT process by regulating circ_0001946/miR-21/GPD1L/HIF-1 alpha pathway		Huang et al. (2013)
	Formononetin	*In vitro*, *in vivo*	U2OS cell	25, 50, 100 mg/kg	Promotes apoptosis by inactivating the intracellular miR-375/ERα-PI3K/Akt cascade pathway		Hu et al. (2019)
cardioprotection	campanulin	*In vivo*	Male Wistar rats	15, 30 mg/kg	Mitigating I/R damage by activating PI3K/Akt signaling pathway		Ren et al. (2016)
	campanulin	*In vivo*	H9C2 cell	30 mg/kg	Activation of the JAK2/STAT3 signaling pathway through upregulation of IL-10 secretion to attenuate myocardial I/R injury		Liu et al. (2020)
	Formononetin	*In vitro*	Senescent tissue, H9C2 cell	5 μM	Mitigating I/R-induced apoptosis in senescent cells by promoting autophagy		Huang et al. (2018b)
	Formononetin	*In vivo*	Rat MIRI model	10, 30 mg/kg	Improved MIRI in rats and inhibited NLRP3 inflammasome activation		Wang et al. (2020a)
	Calycosin	*In vivo*	SD rats	30 mg/kg	Reversed histopathological changes, significantly reduced serum levels of myocardial markers, inhibited inflammatory factors, apoptosis, and oxidative stress		Huang et al. (2020)
	Calycosin	*In vivo*/*in vitro*	H9C2 cell	50, 100, 200 μM	The reduction of apoptosis in H9C2 cells is through regulating Sirt 1-NLRP 3 pathway to cope with DOX-induced cardiotoxicity		Zhai et al. (2020)
	Calycosin	*In vivo*	AIC model	1,10, 50 μmol/L	The formation of autophagic vesicles was promoted by atg7, which improved cardiac function and restored autophagy		Lu et al. (2021)
	Calycosin	*In vivo*/*in vitro*	C57 BL/6 J mice cell	5, 10, 20, 40, 80, 160 μg/mL	It improved DOX-induced myocardial function, inhibited brain natriuretic peptide and improved histomorphological changes in mice		Zhang et al. (2022)
neuroprotection	Formononetin, Calycosin,Campanulin	*In vitro*	PC 12 cell	0.01, 0.05,0.1, 0.5, 1 μg/mL	Addition of endogenous antioxidants decreased the activities of antioxidant enzymes, superoxide dismutase (SOD) and glutathione peroxidase (GSH-PX)		Yu et al. (2009)
	Formononetin	*In vitro*	SD rats	30 mg/kg	The number of dendritic spines and the expressions of NGF, p-AKT, GAP-43, BDNF, p-Trk A, p-Trk B and p-ERK1/2 were significantly increased		Wu et al. (2020)
	Calycosin	*In vivo*	SD rats	5, 10, 20 mg/kg	Estrogen receptor activation, positive feedback regulation of miR-375, dysregulation of RASD1, and upregulation of Bcl-2 leads can reduce brain water content and improve neurological deficits		Hsu et al. (2020)
	Calycosin	*In vivo*	SD rats	5, 10, 20 mg/kg	The levels of P62, NBR1 and Bcl-2 were significantly decreased, and the level of TNF-α was significantly increased		Wang et al. (2014b)
	Formononetin	*In vivo*	SD rats	30, 60 mg/kg	By activating Nrf2/ARE signaling pathway, it inhibits oxidative stress and reduces hippocampal neuronal injury		Yu et al. (2022)
	Calycosin	*In vivo*	SD rats	50 mg/kg	It can inhibit oxidative stress by enhancing the NRF2 antioxidant pathway and inhibit inflammatory response by blocking NACHT, NALP3 and NF-κB pathways		Yao et al. (2022)
	Formononetin, Calycosin,Daidzein	*In vivo*/*in vitro*	Cortical neuronal cells	1, 5, 25 μM	Synergistic activation of estrogen receptor-PI3K-Akt signaling pathway improved neurological deficits and significantly attenuated L-glutamate and OGD/Ro-induced neuronal death		Gu et al. (2021)
antidiabetic	Calycosin	*In vitro*	BRL-3A cells	10^–8^ to 10^–4^ M	Improves AGEs-induced impairment of hepatocyte viability, thereby significantly reducing basal glucose uptake by hepatocytes		Xu et al. (2015)
	Formononetin, Calycosin	*In vivo*	Male mice	0.03–0.1 mg/kg	Enhances the hypoglycemic effect of antifolin in diabetic mice, while relying on antifolin-induced insulin release		Ma et al. (2007)
	Formononetin	*In vivo*	KM mice	5, 10, 20 mg/kg	By inhibiting apoptosis of islet B Cells, promoting regeneration of islet B Cells, promoting insulin secretion, hepatic glycogen synthesis and hepatic glycolysis		Qiu et al. (2016)
	Formononetin	*In vivo*	SD rats	10, 20, 40 mg/kg	Improvements in glucose tolerance, insulin sensitivity, and reduction in lipid levels and glycated hemoglobin levels in blood and significantly increased SIRT-1 expression in pancreatic tissue		Oza and Kulkarni (2018)
anti-oxidant	Formononetin	*In vivo*	BALB/c mice	10, 20, 40 mg/kg	Significantly inhibited the activation of NF-κB and JNK, and significantly increased the expression of heme oxygenase 1 (HO-1)		Cheng et al. (2015)
	Calycosin	*In vitro*	HepG2 cell	8 μg/mL	It significantly inhibited the isoproterenol-induced increase in myeloperoxidase (MPO) activity and malondialdehyde (MDA) levels		Cheng et al. (2015)
	Formononetin	*In vivo*	BALB/c mice	10, 20, 40 mg/kg	Significantly inhibited NF-kB and J n K and increased the expression of heme oxygenase 1 (HO-1)		Yi et al. (2020)
	Calycosin	*in vitro*	Rat brain astrocytes	2.5, 25, 50, 100 μM	Activates signaling pathway, effectively inhibits excessive ROS production and inflammatory factor expression, increases SOD activity, and reduces astrocyte damage		Lu et al. (2022)

### Anti-inflammatory

Inflammation is a defensive response of the body to the external stimuli and is characterized by redness, swelling, fever, pain, and dysfunction. Bacteria such as rickettsiae, mycoplasmas, spirochetes, fungi, and parasites are the most common causes of inflammation (Yang et al., 2021). Modern pharmacology has demonstrated that *Astragalus* isoflavones have anti-inflammatory effects, the main substances of which are FMN and CAL. Yu Ping Feng San (YPFS) is a traditional Chinese medicinal decoction widely used to treat atopic dermatitis (AD). The active ingredients CAL and FMN extracted from YPFS can reduce epidermal thickening at the initial stage of sensitization alone, and they inhibit thymic stromal lymphopoietin (TSLP) by regulating nuclear factor kappa B (NF-κB) activation and translocation, thereby reducing allergic inflammation. This confirms the anti-inflammatory activity of FMN and CAL(Shen et al., 2014).

Numerous anti-inflammatory studies have demonstrated that drugs usually exert their anti-inflammatory effects by modulating the expression of nuclear factors and κB inhibitors such as NF-κB, Interleukin (IL-1β/6/33), tumor necrosis factor (TNF), Mitogen-activated protein kinase (MAPK), thymic stromal lymphopoietin (TSLP), and hypoxia inducible factor-1 (HIF-1α). A study administered FMN to fluorescein isothiocyanate (FITC)-induced AD mice and FITC-treated HaCaT cells followed by polyinosinic: polycytidylic acid or lipopolysaccharide treatment and reported that TSLP/IL-33 levels were reduced *in vitro* and *in vivo* whereas E-calcine mucin levels were increased *in vitro* (Li et al., 2018). This may be because FMN reduces TSLP/IL-33 production while alleviating the inflammatory response by regulating E-calcine mucin. Moreover, FMN can alleviate AD by promoting the upregulation of tumor necrosis factor alpha-inducible protein 3 (A20) expression by siGPER. FMN significantly increases the expression of A20 protein and mRNA while suppressing the expression of TSLP protein and mRNA (Yuan et al., 2021). FMN inhibits the production of inflammatory mediators and cytokines in osteoarthritis (AO), as well as the expression of cyclooxygenase-2 and nitric oxide synthase, thereby inhibiting the synthesis of matrix metalloproteinases (MMPs) and thrombomodulin. This mechanism involves the activation of phosphatases and the inhibition of IL-1β-induced activation of NF-κB and protein kinase B (AKT) (Jia et al., 2022). CAL has been found to ameliorate lung injury and inflammatory response in mice with pneumonia caused by respiratory syncytial virus infection. The mechanism may be related to the inhibition of NF⁃κB signaling pathway activation. CAL acts on AO by inhibiting IL-1β protein-induced activation of PI3K/AKT/FoxO1 signaling (Guo et al., 2022); it can mitigate sepsis-induced acute lung injury through the HMGB1/MyD88/NF-κB pathway and activation of NLRP3 inflammatory vesicles (Chen et al., 2021). HIF-1α may be a therapeutic target in AD when CAL is used to treat AD. CAL can inhibit HIF-1 α expression both *in vivo* and *in vitro*; it downregulates HIF-1 α expression in HaCaT cells to repair tight junctions and reduce allergic inflammation (Jia et al., 2018).

### Anti-tumor

Tumors arise from the proliferation of local tissue cells affected by various tumorigenic factors in the body. Tumors are classified as benign and malignant, and cancer is a type of malignant tumor that originates from epithelial tissue. According to the studies conducted in the last decade, FMN and CAL can treat oncological diseases, including lung (Yang et al., 2014), breast (Yu et al., 2017), colorectal (Hu et al., 2023), ovarian (Yao et al., 2019), and gastric cancers (Zhou et al., 2015), via various molecular pathways. Their mechanism of anti-tumor action includes inhibition of cell proliferation, influence on the cell cycle, and induction of apoptosis.

#### Multiple pathways to inhibit tumor cell proliferation

The four isoflavone extracts of Huangqi, GC, CAL, FMN, and ON, have been found to inhibit the proliferation of SK-BR-3, MCF-7, and MDA-MB-231 cells in a dose-dependent manner, as well as to decrease the levels of p-GS3K β, p-PI3K, p-Akt, and p-mTOR and substantial increase total mTOR levels (Zhou et al., 2018). Additionally, CAL can regulate the circ_0001946/miR-21/GPD1L/HIF-1α signaling axis in a dose-dependent manner. miR-21 is the most recognized and significant miRNA associated with carcinogenesis and is involved in the pathogenesis of many cancers (Huang et al., 2013). CAL downregulates miR-21 at circ_0001946 and GPD1L levels and upregulates HIF-1α levels in lung adenocarcinoma cells, thereby inhibiting cell proliferation, invasion, migration, and epithelial-mesenchymal transitions (EMT) processes (Zhou et al., 2018). Extracellular regulatory protein kinase 1/2 (ERK1/2) can enter the nucleus to promote the transcription and expression of certain genes and is closely related to cell proliferation and differentiation. FMN can act by inhibiting the ERK1/2 pathway and inactivating laminin A/C in nasopharyngeal carcinoma (NPC) cells. Further, B-cell lymphoma-2 (Bcl-2), ERK1/2, laminin A/C, and CK19 expressions have been found to be downregulated in FMN-treated NPC CNE2 cells, whereas intracellular Bax expression is elevated, indicating an inhibition of cell proliferation (Ying et al., 2019).

#### Influence on the cell cycle through multiple pathways

CAL can inhibit breast cancer cell growth by regulating AKT signaling pathway, inducing the activation of MAPK, STAT3, NF-κB, and related apoptotic proteins and reducing the expression levels of TGF-β1, SMAD2/3, and SLUG to arrest the cell cycle in G0/G1 phase (Zhou et al., 2018). CAL inhibits Bcl-2 expression and promotes Bax, caspase-3, PARP, TGF-β1, SMAD2/3, and SLUG expressions by blocking the growth of hepatocellular carcinoma BEL-7402 cell line in G0/G1 phase. In addition, CAL induces MAPK, STAT3, NF-κB, and related apoptotic proteins in HepG2 hepatocellular carcinoma cells by regulating AKT pathway protein activation to induce G0/G1 phase cell cycle arrest (Liu et al., 2021b). Furthermore, FMN inhibits colon cancer (SW1116 and HCT116) cell growth through miR-149-induced downregulation of EphB3 and inhibition of PI3K/AKT and STAT3 signaling pathways to downregulate cell cycle-associated protein Cyclin D1 expression and block the cell cycle at the G0/G1 point (Wang et al., 2018a). FMN induces G1 phase arrest in MCF-7, SK-BR-3, and MDAMB-231 breast cancer cells by downregulating the expression of Cyclin D1 and Cyclin E and negatively regulating the expression of P21 and P27 (Zhou et al., 2016).

#### Induction of apoptosis through multiple pathways

FMN has been shown to alleviate ovarian cancer in SKOV-3 cells by increasing E-cadherin expression and decreasing MMP-9 expression, which inhibits the cancer cell proliferation, migration, and invasion (Gu et al., 2020). Further studies have demonstrated that FMN causes apoptosis in SKOV-3 cells. The anti-tumor effect of FMN is achieved by regulating the miR-19b-3p/TGF-β/Smad2 signaling pathway (Niu et al., 2021). The Bcl-2 protein family significantly inhibits apoptosis, and FMN shows a dose-dependent inhibition of Bax/Bcl-2 and caspase-3/9 protein expressions in ovarian cancer cells, thereby exhibiting anti-proliferative, anti-migratory, and invasive effects. The Bax/Bcl-2 ratio has been found to increase after FMN treatment, whereas caspase-3 and caspase-9 levels are elevated (Lee et al., 2018). CAL induces p21Waf1/Cip1 cycle arrest and promotes caspase apoptosis and MIA PaCa-2 cell migration in macrophages RAW 264.7, which occurs through the induction of the Raf/MEK/ERK pathway and promotion of M2 tumor-associated macrophages acting in the tumor microenvironment (Zhang et al., 2020). CAL can reduce the viability of colorectal cancer (CRC) cells through targeted inhibition of PI3K/Akt signaling pathway and upregulation of Phosphatase gene (PTEN) protein and estrogen receptor *ß* (Erβ), thereby inducing CRC cell apoptosis. PTEN and ERβ protein expressions are significantly upregulated in CRC cells subjected to CAL, whereas p-AKT/AKT ratio and Bcl-2 levels are downregulated, confirming the anti-tumor effect of CAL(Zhang et al., 2021b). FMN induces apoptosis in OGS cells and inhibits the growth of solid tumors, resulting in an increase in intracellular Apaf-1 positive cells and a decrease in endogenous Ki-67, p-PI3KCATyr317, and p-AKTSer473 immune cells. The mechanism of action is related to the inactivation of miR-375/ERα-PI3K/AKT signaling pathway in cells (Hu et al., 2019).

#### Treatment of heart diseases

Cardiovascular diseases account for approximately 17.5 million deaths worldwide annually, it is crucial to screen for effective therapeutic agents against these diseases (Xin et al., 2013). The most common and critical heart diseases include hypertension, coronary artery disease, and arrhythmia, which can occur independently or in combination with other heart diseases. *Astragalus* isoflavones have anti-apoptotic, autophagy-promoting, anti-inflammatory, and antioxidant roles in heart diseases.

CAL can inhibit cardiomyocyte apoptosis by promoting the activation of the PI3K/AKT signaling pathway, thereby reducing myocardial injury. High-dose CG pretreatment has been shown to significantly improve cardiac function in rats, with the upregulation of superoxide dismutase (SOD), Ejection Fraction (EF), fractional shortening (FS), and left ventricular end-systolic pressure and downregulation of left ventricular end-diastolic pressure and malonaldehyde (MDA). Caspase-3 and caspase-9 activities were also inhibited (Ren et al., 2016). Further studies revealed that CG may mitigate ischemia/reperfusion (I/R) injury by upregulating IL-10 to activate the JAK2/STAT3 signaling pathway (Liu et al., 2020). Using isolated heart tissues from senescent mice and chemically induced senescent H9C2 cells as experimental subjects, a study demonstrated that FMN can attenuate I/R-induced apoptosis in cells or tissues (Huang et al., 2018b). Additionally, FMN can inhibit the activation of nod-like receptor protein 3 (NLRP3) inflammasome in rats and improve IR in rats via the reactive oxygen species (ROS)-TXNIP-NLRP3 signaling pathway (Wang et al., 2020a). CAL protects the heart by eliminating histopathological changes owing to its anti-inflammatory, anti-apoptotic, antioxidant, and anti-lipid peroxidation activities. CAL may exert cardioprotective effects by modulating the Sirt1-NLRP3 pathway, thereby ameliorating adriamycin/adriamycin (DOX)-induced cardiotoxicity, reducing apoptosis and inhibiting oxidative stress. CAL may also be useful in the treatment of myocardial infarction to reduce cardiac dysfunction and its associated complications (Huang et al., 2020). CAL induces apoptosis through the Bcl-2, Bax, and PI3K-Akt signaling pathways and increases H9C2 cell viability. In addition, CAL has been shown to improve Sirt1-NLRP3 levels in cells and mouse hearts. CAL can improve cardiac function in adult zebrafish and restore autophagy through atg7 autophagy-mediated production of protection against DOX-induced cardiotoxicity (Lu et al., 2021). Zhang et al. established a cardiotoxicity model using DOX stimulation in H9C2 cells and C57BL/6J mice. The cardioprotective mechanism was confirmed using *in vivo* and *ex vivo* experiments, which showed that CAL alleviated DOX-induced cardiotoxicity by inhibiting the activation of NLRP3 inflammatory vesicles (Zhang et al., 2022).

#### Treatment of neurological diseases

Neurological diseases are pathological conditions that negatively affect the peripheral nervous system, spinal cord, and/or brain, ultimately leading to functional disorders (Gunata et al., 2020; Cao et al., 2023). Its etiology is complex and includes trauma, infection, genetics, tumors, immunology, and several other factors that can lead to neurological dysfunction, resulting in neurological diseases. Several studies have demonstrated the neuroprotective properties of FMN and CAL against cerebral ischemia, dementia, traumatic brain injury, Alzheimer’s disease, anxiety, and depression. The pathways involved in these neuroprotective mechanisms are ERPI3K-Akt, PI3K/AKT/ERK, and ROS-TXNIP-NLRP3 pathways. The neuroprotective effect of five *Astragalus* isoflavone compounds on xanthine (XA)/xanthine oxidase (XO)-induced damage in PC12 cells has been investigated. The reduction of SOD, antioxidant glutathione peroxidase, and enzymatic activities is prevented in isoflavone-treated cells; these neuroprotective effects may be produced by increasing endogenous antioxidants (Yu et al., 2009). FMN can promote the expression of NGF, GAP-43, BDNF, p-Trk A, p-Trk B, p-ERK 1/2, and p-AKT by increasing the number of neuronal dendritic spines and *ß* III-microtubulin, with the best effect at 30 mg/kg (Wu et al., 2020). CAL treatment in stroke patients increases brain BDNF/TrkB expression, ameliorates neurological damage, and transforms microglia from an activated amoeboid state to a resting branching state. BDNF/TrkB -mediated CAL ameliorates ischemic stroke injury in rats by switching microglia from an activated to a resting branching state (Hsu et al., 2020). The pathogenic mechanisms underlying I/R include elevated intracellular Ca^2+^ levels, excitatory neurotransmitter release, oxidative stress, inflammation, and apoptosis (Durukan and Tatlisumak, 2007). CAL has neuroprotective effects in I/R rats, significantly reducing the brain water content and improving neurological deficits. The mechanism of action may be related to the positive feedback regulation of miR-375 through ER-α (Wang et al., 2014b). The effect of CAL on I/R may be related to its anti-autophagic, anti-apoptotic, and anti-inflammatory activities. A study established an I/R rat model with middle cerebral artery occlusion and reported that CAL pretreatment for 14 days significantly reduced brain edema and improved neurological function in I/R rats, as well as significantly upregulated the expression of Bcl-2, p62, and NBR1 and downregulated the level of tumor necrosis factor alpha (TNF-α) (Wang et al., 2018b). FMN action in I/R rats reduces ASC, p-STAT3, p-JAK2, NLRP3, cl-IL-1β, and cl-caspase-1 protein levels in the brain tissue of rats with infarct volume. The neuroprotective effect of FMN is achieved through the inhibition of the JAK2/STAT3 signaling pathway (Yu et al., 2022). FMN reduces hippocampal neuronal damage and oxidative stress in rats, improves depression-like behavior in rats with mild stress (CUMS)-induced depression, and reverses the CUMS-induced decrease in nuclear factor erythroid-2-related factor 2 (Nrf2) protein and increase in NQO⁃1 and HO⁃1 proteins in the nucleus (Yao et al., 2022). CAL may also be effective against cerebral hemorrhage-induced injury by inhibiting oxidative damage and inflammatory responses, and 50 mg/kg CAL has been shown to significantly inhibit ischemic brain injury. Lesion volume, blood volume, and hemispheric enlargement are significantly reduced after CAL treatment. CAL likely inhibits oxidative stress by enhancing the Nrf2 antioxidant pathway and suppresses the inflammatory response by blocking the activation of NACHT, NALP3 inflammatory vesicles, and NF-κB pathway (Chen et al., 2020). *Astragalus* isoflavones alleviate I/R by activating the ER-PI3K-Akt pathway, which may be a molecular target for synergistic neuroprotection by Astragalus isoflavones (Gu et al., 2021).

### Anti-diabetic

Diabetes is a chronic endocrine disease characterized by glucose, fat, and protein metabolism disorders caused by insulin deficiency, insulin insensitivity, or both, which can lead to the damage and dysfunction of various organs in the body (Krasteva et al., 2014). Type 1 diabetes is characterized by absolute insulin deficiency, whereas type 2 diabetes is characterized by relative insulin deficiency and insulin resistance. CAL ameliorates advanced glycation end products (AGEs)-induced impairment of hepatocyte viability and AGEs-induced dysfunction of hepatocyte glucose uptake in a dose-dependent manner (Xu et al., 2015). The combined application of FMN, CAL, and Tetrandrine has been demonstrated to be effective against hyperglycemia and hypoinsulinemia in streptozotocin (STZ)-induced diabetic mice (Ma et al., 2007); this is because FMN and CAL can enhance the hypoglycemic effect of Tetrandrine. In another study, FMN significantly reduced the fasting blood glucose levels at doses of 5, 10, and 20 mg/kg in alloxan-induced type 1 diabetes mice, indicating that FMN promotes islet B cell regeneration, insulin secretion, and liver glycogen synthesis by inhibiting islet B cell apoptosis (Qiu et al., 2016). FMN can also treat STZ-induced type 2 diabetes. Significant improvement in the fasting blood glucose levels has been observed after 40 mg/kg FMN treatment of rats, and FMN at doses of 10, 20, and 40 mg/kg can significantly reduce serum urea nitrogen, glucose, albumin, and creatinine levels. Further, FMN can significantly increase lipid peroxidation and SOD levels and reduce renal peroxidase activity, cytokine levels, inflammatory changes, and renal cell necrosis, thereby protecting pancreatic *ß*-cells from necrosis and degeneration (Jain et al., 2020).

### Anti-oxidant

Recent studies have reported that Huangqi extract has strong antioxidant activity and may act as a free radical scavenger, thereby alleviating the symptoms of oxidative stress in the early stages of diabetic nephropathy. Some studies have discovered that CAL and GC have significant anti-lipid peroxidation activity (Kim et al., 2003). FMN, CAL, and CA, isolated from Huangqi, have been found to significantly inhibit XA/XO-induced cell damage; they have significant superoxide anion and free radical (DPPH) radical scavenging abilities, which can effectively inhibit cell damage caused by XA and XO. Among these compounds, CAL has the most prominent antioxidant activity (Yu et al., 2005). Studies have reported that Huangqi extract can improve blood lipid levels, inhibit lipid peroxidation, increase the activity of antioxidant enzymes, and reduce the risk of hyperlipidemia and oxidative stress-related coronary heart disease in humans (Ma et al., 2011). In addition, the combination of CAL with gallic acid can significantly inhibit increased myeloperoxidase (MPO) activity due to isoproterenol (ISO) (Cheng et al., 2015). Oxidative stress-induced brain cell damage is an important factor in the pathogenesis of ROS-related nervous system diseases. Astrocytes are important immunocompetent brain cells that play a role in various nervous system diseases. CAL regulates oxidative stress through the AKT/Nrf2/HO-1 pathway, thereby preventing oxidative damage in brain astrocytes. CAL-treated cells exhibit enhanced viability, inhibition of ROS and inflammatory factor production, increased SOD expression, and dose-dependent inhibition of H_2_O_2_-induced damage (Lu et al., 2022). CAL was found to exert antioxidant effects by restoring SOD/CAT activity and reducing ROS content and caspase-3 activity in a Parkinson’s disease model, thereby altering α-syn amyloid-induced neurotoxicity (Pan et al., 2021). In an allergic asthma model, treatment with FMN (10, 20, and 40 mg/kg) and the positive control drug dexamethasone (2 mg/kg) decreased ROS activity and increased SOD activity increased. The oxidation-related signaling molecules involved in this action are c-Jun N-terminal kinase (JNK), NF-κB, and the transcription factor Nrf2(Yi et al., 2020).

### Other pharmacological effects

*Astragalus* isoflavones also exhibit antiviral, estrogen-like, antibacterial, hepatoprotective, and immune-enhancing effects. Isoflavones have a molecular structure similar to that of estrogen and can therefore bind to estrogen receptors; hence, they are classified as phytoestrogens. CAL has a protective effect on the liver of mice with acute immune liver injury caused by concanavalin A (ConA) (Liang et al., 2018), likely because of its antioxidant effect on free radicals and the enhancement of estrogen-like effects by promoting hepatic ER expression. FMN may enhance estrogen-like effects by promoting estrogen receptor protein expression (El-Bakoush and Olajide, 2018) and exert antimicrobial effects by attenuating the cytotoxic and inflammatory response of *Streptococcus suis in vitro*; lysozyme could be an ideal target against this pathogen (Wang et al., 2020b). Further, FMN has anti-apoptotic and anti-inflammatory effects on the liver mainly by inhibiting the expression of TNF-α, NF-κB-p65, TLR3, and NLRP3 and upregulating Bcl-2. It also exerts anti-metabolism-related effects on fatty liver disease through lipophagy (Liu et al., 2021a). CAL exhibits hepatoprotective functions mainly by affecting the expression of STAT3, FXR, a-SMA, and ERβ5, which in turn regulates free fatty acid *ß*-oxidation, gluconeogenesis, triglyceride synthesis, glucose metabolism, collagen deposition, and hydroxyproline content (Duan et al., 2017; Duan et al., 2018).

### Toxicology

Although *Astragalus* has been widely used in clinical practice for several years, comprehensive safety and toxicity assessments have not yet been conducted. Studies on the toxicology of *Ast*ragalus have long been of interest to researchers, especially those focusing on the therapeutic toxicity of secondary metabolites of *Astragalus*. *Astragalus* species can be classified into three main categories based on their toxic effects on animals: species that can synthesize aliphatic nitro compounds, species that can cause madder poisoning, and species that can accumulate selenium (Rios and Waterman, 1997). Toxicological studies on astragaloside have shown that it is toxic above a dose of 1.0 mL/kg to some embryos as well as mothers. However, no specific toxicities such as acute toxicity, subacute or subchronic toxicity, genotoxicity, or immunotoxicity have been observed (Jiangbo et al., 2009). The extract of *Astragalus*, historically recognized as a traditional medicine and food, has now been evaluated for its subchronic toxicity and genotoxic safety as a modern dietary ingredient, along with the triterpene glycosidic cyclic element astragalinol (Szabo, 2014). Rats were administered astragalol at 0, 40, 80, and 150 mg/kg/day for 91 consecutive days, but no treatment-related deaths or cardiac effects were observed. In a toxicity study on Huangqi extract, acute and subchronic oral toxicity tests were performed on rats. In acute toxicity studies, a single dose can reach up to 5,000 mg/kg. In a 13-week subchronic toxicity study based on clinical symptoms, body weight, and autopsy results, there were no deaths or toxic reactions (Song et al., 2017). Huangqi can be used for food health and consumed for a long time at standard doses. Because *Astragalus* species may be contaminated with pesticides or heavy metals during cultivation, leading to increased safety concerns (Jiaojiao et al., 2019) and reducing its value, it is important to control the use of pesticides and conduct soil quality testing.

In addition, the combination of Huangqi can achieve the effect of increasing efficiency and reducing toxicity. Apatinib mesylate combined with astragaloside can significantly inhibit the growth of hepatocellular carcinoma transplantation tumors in nude mice, promote the apoptosis of transplantation tumor cells, and cause inhibitory effects on the proliferation, migration, and invasion of HCC cells (Sun et al., 2023). Astragaloside (Peng et al., 2017), astragaloside (Lina et al., 2022) and doxorubicin can alleviate cardiotoxicity and improve anti-tumor effect. The combination of astragaloside and angiotensin-converting enzyme inhibitors (ACEi) can reduce the degree of proteinuria and delay the progression of diabetic kidney injury in mice (Li et al., 2021). The above experimental results indicate that the combination of Huangqi active ingredients with other drugs has shown certain advantages in basic research, but the mechanism of its efficiency and toxicity reduction needs to continue to be explored. Although the safety of Huangqi has been heavily formalized, independent studies on Huangqi are still lacking, and further *in vitro* and clinical trials are required for confirmation.

## Quality control

### Analysis methods

Misidentification and adulteration of varieties are the main problems in the identification of herbal medicines (Zhu et al., 2022). Due to non-standard market systems and market supervision and control, counterfeit and inferior Huangqi products often appear. The quality of Huangqi medicinal herbs is also influenced by different geographical locations, cultivation techniques, and climatic environments (Yang et al., 2020). Therefore, the key to the quality control of Active ingredients in Huangqi lies in the establishment of quality analysis methods.

At present, the 2020 edition of the Chinese Pharmacopoeia controls the quality of Huangqi from three aspects: morphology, microscopy, and thin-layer chromatography. It is required that the moisture content shall not exceed 10.0%, the total ash content shall not exceed 5.0%, and the leaching content shall not be less than 17.0% (Gong et al., 2018). The content of astragaloside A determined by High-performance liquid chromatography shall not be less than 0.080%, and the content of calyx Isoflavone glucoside shall not be less than 0.020%. Traditionally, High-performance liquid chromatography was used to determine the content of Huangqi. However, these methods may not be sufficient to evaluate the quality of Huangqi medicinal herbs. With the improvement of analytical technology, people have adopted other methods to determine the chemical composition of Huangqi and control its quality. For example, chromatography-mass spectrometry (LC-MS), external spectroscopy (IR), and ultraviolet spectroscopy (UV) provide effective means for quantitative analysis of the Active ingredients of Huangqi (Huang et al., 2018a).

### Chemical fingerprint

Traditional Chinese medicine fingerprint is a comprehensive and quantifiable identification method established based on systematic research on the chemical composition of traditional Chinese medicine, used to evaluate the authenticity, stability, consistency, and effectiveness of traditional Chinese medicine. The fingerprint of traditional Chinese medicine, as a standard for quality control, has also been included in the Chinese Pharmacopoeia. Currently, the Chinese Pharmacopoeia does not include the fingerprint of Huangqi. Scholars (Wang et al., 2023) have established a UPLC fingerprint and content determination method for the stem and leaf of Mongolian Huangqi, and compared and analyzed 15 batches of Mongolian Huangqi stem and leaf samples from different regions. A rapid and effective method for evaluating the quality of *Astragalus mongholicu* stem and leaf has been established. DNA barcoding technology also shows broad application prospects in the identification of astragalus medicinal materials. The fingerprint determined by LC-MS combined with the ITS interval domain DNA map uses the astragalus plant genome region as the barcode, which can quickly and accurately classify the source plants, and can be used as the barcode mark for quality control of astragalus (Xiao et al., 2011). With the development of technology, quality marker (Q-marker) was proposed in 2016 (Changxiao et al., 2016), and the idea of an “effect component target fingerprint” was discovered (Liao et al., 2018) to predict and identify the quality of Chinese medicine Q-marker through network pharmacology and high-performance liquid chromatography fingerprint. Li et al. (Li et al., 2022) established a reliable analytical method combined with network pharmacology, established fingerprint spectra of 23 batches of Huangqi, successfully isolated and quantified 8 compounds, and is expected to become a new approach for quality control of Huangqi (Huang et al., 2018a). At present, the HPLC-ELSD method is mainly used for the development of fingerprints of saponins and polysaccharides; The HPLC-DAD/HPLC-UV method can be used to establish the fingerprint of flavonoids and polysaccharides.; The PLC-CAD method can be used to develop fingerprints of flavonoids and saponins (Zhen et al., 2023).

With the continuous development of technology, the quality control methods of medicinal materials are also constantly innovating. With the discovery of the pharmacological effects of the Active ingredient of Astragalus, the innovation of quality control methods and technologies of Astragalus is becoming more and more important. These methods and technologies are convenient for people to understand Chinese medicinal materials truly, quickly, and accurately, and provide a reference and basis for the quality control of the Active ingredient of Huangqi.

## Conclusion and future perspectives

This review discusses the recent advances in botany, phytochemistry, traditional uses, pharmacology, toxicology, and quality control of *Astragalus*. Presently, several pharmacological studies have been conducted on *Astragalus* isoflavones, including FMN and CAL. Therefore, this review mainly focused on the pharmacological effects of isoflavones. Pharmacological studies have shown that isoflavones possess many pharmacological activities, including anti-inflammatory, anti-tumor, anti-diabetic, cardioprotective, neuroprotective, and antioxidant effects. They are also used in many other applications aspects to their diverse activities. However, despite extensive pharmacological research on *Astragalus* isoflavones, some problems require further discussion.

First, *Astragalus* has a large demand as a medicine as well food; therefore, its clinical application should be extensively investigated to avoid excessive dosage and incompatibility. Meanwhile, herbs such as Huangqi are highly popular in both China and abroad. Tea, soup, and congee have become important media for healthcare, with the research and production of healthcare products increasing. Additionally, it has been established that Huangqi has a wide range of applications in herbal healthcare, particularly in immunomodulation and regulation of blood glucose. According to the clinical pharmacological effects of Huangqi, the use of Huangqi in combination with other herbal medicines in healthcare products should not be limited to the above functions but can also be studied in the areas of auxiliary improvement of memory, sleep, growth, and development, promotion of digestion, and auxiliary protection against gastric mucosa damage.

Second, more than 200 compounds have been isolated from *Astragalus* species; although flavonoids and saponins have been comprehensively studied, the study of polysaccharide components of *Astragalus* remains limited. Some studies show that highly valuable the field of pharmacology is; in fact, pharmacological research and clinical applications are inseparable; therefore, combining pharmacological and clinical studies makes the application of Huangqi possible in many fields.

Third, in terms of pharmacological effects, recent studies on the active ingredients of *Astragalus* have mainly focused on FMN and CA, and studies on other active compounds and their effects are limited. In addition, most of these studies have focused on the anti-tumor and anti-inflammatory effects of FMN and CAL; however, the mechanism and target of action of the main pharmacological effects, such as anti-tumor and anti-inflammatory effects, are not fully understood. The number of samples was small, the type was single, and the pathological characteristics of different clinical patients were considered and studied. Future pharmacological research should focus on exploring active ingredients and their mechanisms of interaction with specific target ingredients, which can lay the foundation for expanding clinical applications in the future while also providing modern pharmacological interpretations of traditional applications.

Fourth, embryotoxicity and maternal toxicity have been observed above 1.0 mL/kg *Astragalus* methoside administration. However, the dose–effect relationship between the safety and toxicity of isoflavones, a phytoestrogen of *Astragalus*, has not been studied. Thus, the mechanism of action and toxicological properties of *Astragalus* require further investigation. *Astragalus* may be contaminated by pesticides and heavy metals during cultivation, leading to increased safety problems and reduced value; therefore, there is a need to control the use of pesticides and conduct soil quality tests.

In conclusion, traditional Chinese medicine, Huangqi, has a wide range of medicinal properties. In this review, we discuss the research progress on the botanical features, phytochemistry, traditional applications, pharmacology, toxicology, and quality control of *Astragalus*. This information can lay a theoretical foundation for the future development and new clinical applications of Huangqi.

## Figures and Tables

**Table:** 

**Huangqi**	**A**stragali Radix
**FMN**	**f**ormononetin
**CAL**	**c**alycosin
**CG**	**c**alycosin-7-glucoside
**ON**	**o**nonin
**ASP**	**p**olysaccharide
**A20**	**t**umor necrosis factor alpha-inducible protein 3
**AD**	**a**topic dermatitis
**ER**	**e**strogen receptor
**ER α**	**e**strogen receptor alpha
**ER β**	**e**strogen receptor β
**FITC**	**f**luorescein isothiocyanate
**HIF-1 alpha**	**h**ypoxia-inducible factor alpha
**IL-1β/6/33**	**i**nterleukin
**siRNA**	**s**mall interfering RNA
**siA20**	**s**mall interfering RNA
**siGPER**	**s**mall interfering RNA
**TNF-α**	**t**umor necrosis factor alpha
**TNF**	**t**umor necrosis factor
**TSLP**	**t**hymic stromal lymphopoietin
**YPFS**	**Y**u Ping Feng San
**EMT**	**e**pithelial-mesenchymal transitions
**MAPK**	**m**itogen-activated protein kinase
**PTEN**	**p**hosphatase gene
**EF**	**E**jection Fraction
**AGEs**	**a**dvanced glycation end products
**ROS**	**r**eactive oxygen species
**anti-SARS-CoV2**	**n**ovel coronavirus
**MMPs**	**m**atrix metalloproteinases
**IL-1β**	**I**nterleukin
**LPS**	**l**ipopolysaccharide
**Bcl-2**	**B**-cell lymphoma-2
**ERK1/2**	**e**xtracellular regulatory protein kinase 1/2
**SOD**	**s**uperoxide dismutase
**LVESP**	**l**eft ventricular end-systolic pressure
**ISO**	**i**soproterenol
**DOX**	**a**driamycin
**MDA**	**m**alondialdehyde
**CUMS**	**m**ild stress
**AGEs**	**g**lycation end products
**INS**	**i**nsulin
**STZ**	**s**treptozotocin
**α-SYN**	**α**-synuclein
**I/R**	**i**schemia/reperfusion
**RSV**	**r**espiratory syncytial virus
**AO**	**o**steoarthritis
**CLP**	**a**ppendiceal ligation puncture
**ALI**	**a**cute lung injury
**NPC**	**n**asopharyngeal carcinoma
**HIF-1α**	**h**ypoxia inducible factor-1
**MDA**	**m**alonaldehyde
**FS**	**f**ractional shortening
**DPPH**	**f**ree radical
**ConA**	**c**oncanavalin A
**Q-maker**	**q**uality markers
