# Supplementary material for: A review of the botany, phytochemistry, traditional uses, pharmacology, toxicology, and quality control of the Astragalus memeranaceus
Source: Front Pharmacol. 2023 Aug 23;14:1242318. doi: 10.3389/fphar.2023.1242318 (PMC10482111; doi:10.3389/fphar.2023.1242318)
Supplement: Supplementary file 1 [file Table1.docx]

**TABLE 1 Flavonoids isolated from Huangqi.**

| No. | Name | [Molecular](https://cn.bing.com/dict/search?q=molecular&FORM=BDVSP6&cc=cn) [formula](https://cn.bing.com/dict/search?q=formula&FORM=BDVSP6&cc=cn) | Parent nucleus | Substituent | Ref |
| --- | --- | --- | --- | --- | --- |
| 1 | [Oroxylin-A](https://www.chemsrc.com/en/cas/480-11-5_260156.html) | C_16_H_12_O_5_ |  | R_1_=H, R_2_=OMe | (Hua et al., 2016) |
| 2 | Wogonin | C_16_H_12_O_5_ |  | R_1_=OMe, R_2_=H | (Hua et al., 2016) |
| 3 | 4',7-dihydroxyflavone | C_15_H_10_O_4_ |  | R=H | (Li et al., 2017b) |
| 4 | 3',4',7‐trihydroxy flavone | C_15_H_10_O_5_ |  | R=OH | (Li et al., 2017b) |
| 5 | (-)-[Liquiritigenin](https://www.chemsrc.com/cas/578-86-9_830446.html) | C_15_H_12_O_4_ |  |  | (Li et al., 2017b) |
| 6 | Quercetin | C_15_H_10_O_7_ |  | R=H | (Wang et al., 2015) |
| 7 | Quercetin 3‐O‐β‐D‐glucopyranoside | C_21_H_20_O_12_ |  | R=Glc | (Wang et al., 2015) |
| 8 | Rhamnocitrin 3‐O‐β‐D‐glucopyranoside | C_28_H_32_O_16_ |  | R=H | (Hao et al., 2016) |
| 9 | Complanatuside | C_28_H_32_O_16_ |  | R=Glc | (Hao et al., 2016) |
| 10 | Astraflavonoid A | C_36_H_36_O_18_ |  | R=5-O-[(E)-p-feruloyl]-Api(f)-(1→2)-Glc | (Hao et al., 2016) |
| 11 | Kaempferol | C_15_H_10_O_6_ |  | R=H | (Hao et al., 2016) |
| 12 | Kaempferol 3‐O‐β‐D‐glucoside | C_21_H_20_O_11_ |  | R=Glc | (Hao et al., 2016) |
| 13 | Kaempferol 3‐O‐(2‐O‐α‐L‐rhamnopyranosyl)‐β‐D‐glucopyranoside | C_27_H_30_O_16_ |  | R=Rha-(1→2)-Glc | (Hao et al., 2016) |
| 14 | Rhamnocitrin 3‐O‐neohesperoside | C_28_H_32_O_15_ |  | R_1_=Rha-(1→2)-Glc, R_2_=Me | (Hao et al., 2016) |
| 15 | Kaempferol 3,7‐di‐O‐β‐D‐glucopyranoside | C_27_H_30_O_16_ |  | R_1_=Glc, R_2_=Glc | (Hao et al., 2016) |
| 16 | Astraflavonoid B | C_43_H_48_O_23_ |  | R_1_=6-O-[(E)-*p*-feruloyl]-Glc, R_2_=Rha-(1→2)-Glc | (Hao et al., 2016) |
| 17 | 4'‐hydroxyflavanone 7‐O‐β‐D‐glucoside | C_21_H_22_O_9_ |  |  | (Li et al., 2014) |
| 18 | Rhamnocitrin 3‐O‐β‐D‐glucopyranoside (1 → 2) ‐β‐D‐apiofuranosyl | C_27_H_30_O_15_ |  | R=Api(f)-(1→2)-Glc | (Wang et al., 2017) |
| 19 | Rhamnocitrin 3‐O‐β‐neohesperidoside | C_28_H_32_O_16_ |  | R=Rha-(1→2)-Glc | (Wang et al., 2017) |
| 20 | Tiliroside | C_30_H_26_O_13_ |  | R_1_=6-O-[(E)-p-coumaroyl]-Glc, R_2_=H | (Wang et al., 2017) |
| 21 | Isorhamnetin 3‐O‐(6‐O‐α‐L‐fucopyranosyl) ‐β‐D‐glucopyranoside | C_28_H_32_O_16_ |  | R_1_=Fuco-(1→6)-Glc, R_2_=OMe | (Wu et al., 2015) |
| 22 | Isorhamnetin 3‐O‐(6‐O‐α‐L‐fucopyranosyl) ‐β‐ D‐galactopyranoside | C_28_H_32_O_16_ |  | R_1_=Fuco-(1→6)-Gal, R_2_=OMe | (Wu et al., 2015) |
| 23 | Isorhamnetin 3‐O‐(4‐O‐[E]‐coumaroyl‐3,6‐α‐L‐O‐fucopyranosyl) ‐β‐D-galactopyranoside | C_43_H_48_O_22_ |  | R_1_=[Fuco-(1→6)-Fuco-(1→3)]-4-O-[(E)-p-coumaroyl]-Gal, R_2_=OMe | (Wu et al., 2015) |
| 24 | Isorhamnetin | C_16_H_12_O_7_ |  | R_1_=H, R_2_=Me | (Bian and LI, 2008) |
| 25 | Quercetin3‐O‐β‐D‐ neospheroside (Rutin) | C_27_H_30_O_16_ |  | R_1_=Rha-(1→2)-Glc, R_2_=H | (Bian and LI, 2008) |
| 26 | Isorhamnetin3‐O‐β‐D‐glucoside | C_22_H_22_O_12_ |  | R_1_=Glc, R_2_=Me | (Bi et al., 2007) |
| 27 | Kaempferol 4'‐methoxyl‐3‐O‐β‐D‐glucoside | C_22_H_22_O_11_ |  |  | (Bi et al., 2007) |
| 28 | 6''‐Acetyl‐ononin | C_24_H_24_O_10_ |  | R=H | (Zhang et al., 2011) |
| 29 | Formononetin | C_16_H_12_O_4_ |  | R=Glc | (Bian et al., 2006) |
| 30 | Formononetin 7‐O‐β‐D‐glucoside (Ononin) | C_22_H_22_O_9_ |  | R=6-O-Ac-Glc | (Bian et al., 2006) |
| 31 | Calycosin | C_16_H_12_O_5_ |  | R=H | (Bian et al., 2006) |
| 32 | Calycosin 7‐O‐β‐D‐glucopyranoside | C_22_H_22_O_10_ |  | R=Glc | (Zhang et al., 2011) |
| 33 | Calycosin 7‐O‐β‐D‐(6‐O‐acetyl) ‐glucopyranoside | C_24_H_24_O_11_ |  | R=6-O-Ac-Glc | (Zhang et al., 2011) |
| 34 | 3'‐methoxy‐5'‐hydroxy‐isoflavone 7‐O‐β‐D‐glucoside | C_22_H_22_O_10_ |  |  | (Wen et al., 2010) |
| 35 | Genistein | C_15_H_10_O_5_ |  | R=H | (Wang et al., 2017) |
| 36 | Genistin | C_21_H_20_O_10_ |  | R=Glc | (Wang et al., 2017) |
| 37 | Glycitein | C_16_H_12_O_5_ |  | R=H | (Wang et al., 2017) |
| 38 | Glycitin | C_22_H_22_O_10_ |  | R=Glc | (Wang et al., 2017) |
| 39 | Pratensein | C_16_H_12_O_6_ |  | R_1_=H, R_2_=OH, R_3_=OH | (Li et al., 2017b) |
| 40 | Odoratin 7‐O‐β‐D‐glucopyranoside | C_23_H_24_O_11_ |  | R_1_=H, R_2_=OMe, R_3_=Glc | (Hao et al., 2016) |
| 41 | (-)-Methylinissolin 3-O-β-D-{6'‐O‐[(E)‐but‐2‐enoyl]}-glucoside | C_27_H_30_O_11_ |  | R_1_=H, R_2_=H | (Zhang et al., 2011) |
| 42 | Vesticarpan | C_16_H_14_O_5_ |  | R_1_=Glc, R_2_=H | (Zhang et al., 2011) |
| 43 | Licoagroside D | C_22_H_24_O_10_ |  | R_1_=6-O-[(E)-but-2-enoyl]-Glc, R_2_= Me | (Zhang et al., 2011) |
| 44 | 3',7,8- trihydroxy-4-methoxy isoflavone | C_16_H_12_O_6_ |  | R_1_=OH, R_2_=H, | (Li et al., 2017b) |
| 45 | 8,3-dihydroxy-7,4'-dimethoxy isoflavone | C_17_H_14_O_6_ |  | R_1_=OH, R_2_=OMe, | (Su et al., 2021) |
| 46 | Calycosin 7-O-β-D-{6''-[(E)-but-2-enoyl]}-glucopyranoside | C_17_H_14_O_6_ |  | R_1_=H, R_2_=6-O-[(E)-but-2-enoyl]-Glc | (Zhang et al., 2011) |
| 47 | 7,3'-dihydroxy-8,4'-dimethoxy isoflavone | C_26_H_26_O_11_ |  | R_1_= OMe, R_2_= H | (Su et al., 2021) |
| 48 | 3',7-dihydroxy-5'-methoxyisoflavone | C_16_H_12_O_5_ |  |  | (Wen et al., 2010) |
| 49 | 4',5,7-Trihydroxy-3'- methoxyisoflavone | C_16_H_12_O_6_ |  |  | (Yazhou et al., 2012) |
| 50 | Ammopiptanoside A | C_26_H_26_O_10_ |  | R=6-O-[(E)-but-2-enoyl]-Glc | (Zhang et al., 2011) |
| 51 | 4',7-Dihydroxy-3'-methoxy isoflavone | C_16_H_12_O_5_ |  |  | (Wang et al., 2017)s |
| 52 | Afrormosin | C_17_H_14_O_5_ |  | R=H | (Bian et al., 2006) |
| 53 | Odoratin | C_17_H_14_0_6_ |  | R=OH | (Bi et al., 2007) |
| 54 | (-)-methylnissolin | C_17_H_16_O_5_ |  | R=H | (Zhang et al., 2011) |
| 55 | (6aR,11aR)-3,9,10-Tri-methoxypterocarpan | C_18_H_18_O_5_ |  | R=Me | (Subarnas A, 1991) |
| 56 | (-)-Methylinissolin 3-O-β-D-glucoside | C_23_H_26_O_10_ |  | R=Glc | (Zhang et al., 2011) |
| 57 | (-)-Methylinissolin 3-O-β-D-(6'-acetyl)-glucoside | C_25_H_28_O_11_ |  | R=6-O-Ac-Glc | (Zhang et al., 2011) |
| 58 | Pratensein 7-O-β-D-glucopyranoside | C_22_H_22_O_11_ |  | R_1_=OGlc, R_2_=OH, R_3_=Me | (Su et al., 2021) |
| 59 | Sissotrin | C_22_H_22_O_10_ |  | R_1_=OGlc, R_2_=H, R_3_=Me | (Yazhou et al., 2012) |
| 60 | Sophorabioside | C_27_H_30_O_14_ |  | R_1_=H, R_2_=H, R_3_=Rha-(1→2)-Glc | (Hao et al., 2016) |
| 61 | Trifolinhizin | C_22_H_22_O_10_ |  |  | (Su et al., 2021) |
| 62 | Daidzein | C_15_H_12_O_4_ |  |  | (Su et al., 2021) |
| 63 | (3R)-8,2'-Dihydroxy-7,4'-dimethoxyisoflavan | C_17_H_18_O_5_ |  |  | (Bian et al., 2006) |
| 64 | Pendulone | C_17_H_16_O_6_ |  |  | (Zhang et al., 2011) |
| 65 | Isomucronulatol | C_17_H_18_O_5_ |  | R_1_=H, R_2_=H | (Zhang et al., 2011) |
| 66 | 7-O-methylisomucronulatol | C_18_H_20_O_5_ |  | R_1_= H, R_2_= Me | (Subarnas A, 1991) |
| 67 | Isomucronulatol-7-β-O-glucoside | C_23_H_28_O_10_ |  | R_1_= OH, R_2_= Glc | (Zhang et al., 2011) |
| 68 | lsomucronulatol 7,2'-di-β-O-glucoside | C_29_H_38_O_15_ |  | R_1_=Glc, R_2_= Glc | (Subarnas A, 1991)s |
| 69 | (3R)-7,2',3'-Trihydroxy-4'-methoxy-isoflavane | C_16_H_16_O_5_ |  | R_1_=H, R_2_=H, R_3_=H | (Su et al., 2021) |
| 70 | (R)-3-(5-Hydroxy-2,3,4-trimethoxyphenyl)-chroman-7-ol | C_18_H_20_O_6_ |  | R_1_=OH, R_2_=Me, R_3_=Me | (Su et al., 2021) |
| 71 | Sphaerophyside SB | C_23_H_28_O_10_ |  | R_1_=H, R_2_=Me, R_3_=Glc | (Su et al., 2021) |
| 72 | 3'-Hydroxy-24-dimethoxyisoflavan 6-O-β-D -glucopyranoside | C_23_H_28_O_11_ |  |  | (Bian et al., 2006) |
| 73 | (3R) - (-)-Mucronulatol 7-O-β-D-glucoside | C_23_H_28_O_10_ |  | R_1_=Me, R_2_=H, R_3_=Glc | (He and Wang, 1990) |
| 74 | 6''-O-Acetyl-(3R)-2'-hydroxy-3',4'-dimethoyl-isoflavan 7-O-β-D-glucopyranoside | C_25_H_30_O_11_ |  | R_1_=H, R_2_=Me, R_3_=6-O-Ac-Glc | (Yazhou et al., 2012) |
| 75 | Astraflavonoid C | C_23_H_28_O_11_ |  |  | (Hao et al., 2016) |
| 76 | 3,2'-Dihydroxyl-3',4-methoxyisoflavanone 7-O-β-D-glucoside | C_23_H_28_O_11_ |  |  | (Li et al., 2014) |
| 77 | Echinatin | C_16_H_14_O_4_ |  |  | (Li et al., 2017b) |
| 78 | Licochalcone B | C_16_H_14_O_5_ |  |  | (Li et al., 2017b) |
| 79 | (3R,4R)-4,7-Hydroxy-2',3'-dimethoxyisoflavane 4'-O-β-D-glucoside | C_23_H_28_O_11_ |  |  | (Su et al., 2021) |
| 80 | 2',5'-Dicarbonyl-3',4'-dimethoxyisoflavanequinone 7-O-β-D-glucoside | C_23_H_26_O_11_ |  |  | (Su et al., 2021) |
| 81 | 4,4',6'-Trihydroxychalcone | C_15_H_12_O_4_ |  | R=H | (Li et al., 2014) |
| 82 | 4-Methoxy-4',6'-dihydroxychalcone | C_16_H_14_O_4_ |  | R=Me | (Li et al., 2014) |
| 83 | 2',4,4'-Trihydroxychalcone | C_15_H_12_O_4_ |  | R_1_=H, R_2_=H | (Li et al., 2017b) |
| 84 | 2'-Methoxyisoliquiritigenin | C_16_H_14_O_4_ |  | R_1_=OH, R_2_=Me | (Li et al., 2017b) |
| 85 | 4,4'-Dimethyl-6'-hydroxy chalcone | C_17_H_16_O_2_ |  |  | (Li et al., 2014) |
| 86 | Sophorophenolone | C_16_H_10_O_6_ |  |  | (Su et al., 2021) |
| 87 | β‐Sitosterol | C_29_H_50_O |  | R=H | (He and Wang, 1990) |
| 88 | β‐Daucosterol | C_35_H_60_O_6_ |  | R=Glc | (Wang et al., 2014a) |
| 89 | (+)‐Syringaresinol‐O‐β‐D‐glucoside | C_28_H_26_O_13_ |  |  | (Su et al., 2021) |
| 90 | Guanosine | C_10_H_14_N_6_O_4_ |  |  | (Wang et al., 2014a) |
| 91 | Dehydrodiconiferyl alcohol 4, γ'‐di‐O‐β‐D‐glucopyranoside | C_32_H_42_O_16_ |  |  | (Su et al., 2021) |
| 92 | Astramemoside A | C_18_H_22_O_11_ |  |  | (Hao et al., 2016) |
| 93 | α‐DDB (Dimethyl 4,4'‐dimethoxy-5,6,5',6'‐di(methylenedioxy) biphenyl‐2,2'‐dicarboxylate) | C_20_H_18_O_10_ |  |  | (He and Wang, 1990) |
| 94 | Adenosine | C_10_H_13_N_5_O_4_ |  |  | (Su et al., 2021) |
| 95 | 3‐(β‐D‐Ribofuranosyl) ‐2,3‐dihydro‐6H‐  1,3‐oxazine‐2,6‐dione | C_9_H_11_NO_7_ |  |  | (Su et al., 2021) |
| 96 | Adenine | C_5_H_5_N_5_ |  |  | (Wang et al., 2014a) |
| 97 | Uridine | C_9_H_12_N_2_O_6_ |  |  | (Wang et al., 2014a) |
| 98 | Emodin | C_15_H_10_O_5_ |  |  | (Su et al., 2021) |
| 99 | 2,6‐Dimethoxy‐4‐hydroxyphenyl‐1‐O-β‐D‐glucopyranoside | C_14_H_20_O_9_ |  |  | (Hao et al., 2016) |
| 100 | 4‐Hydroxycinnamic acid | C_9_H_8_O_3_ |  |  | (Li et al., 2017b) |
| 101 | Gallic acid | C_7_H_6_O_5_ |  |  | (Su et al., 2021) |
| 102 | Gentisin | C_14_H_10_O_5_ |  |  | (Su et al., 2021) |
| 103 | Maltol‐β‐D‐glucopyranoside | C_12_H_16_O_8_ |  |  | (Su et al., 2021) |
| 104 | Benzyl‐α‐L‐arabinopyranosyl (1'' → 6') ‐β‐D‐glucopyranoside | C_18_H_26_O_10_ |  | R=Ara-(1-6)-Glc | (Hao et al., 2016) |
| 105 | 1‐Hydroxy‐5‐methylolbenzol‐2‐O‐β‐D-glucoside | C_13_H_18_O_8_ |  |  | (Su et al., 2021) |
| 106 | D‐3‐Methoxy‐chior‐inositol | C_7_H_14_O_6_ |  |  | (Hua et al., 2016) |
| 107 | γ‐Aminobutanoic acid | C_4_H_9_NO_2_ |  |  | (He and Wang, 1990) |
| 108 | Palmitic acid | C_16_H_32_O_2_ |  |  | (He and Wang, 1990) |
| 109 | Monopalmitin | C_19_H_38_O_4_ |  |  | (Wen et al., 2010) |
| 110 | L‐Asparagine | C_4_H_8_N_2_O_3_ |  |  | (He and Wang, 1990) |
| 111 | Chrysoeriol-7-O-D-glucopyranosyl-4′-O-α-L-rhamnopyranoside | C_28_H_32_O_15_ |  | R_1_=α-L-Rha, R_2_=OMe, R_3_=H, R_4_=β-D-Glc | (Kavtaradze et al., 2020) |
| 112 | Chrysoeriol-4′-*O*-α-*L*-rhamnopyranoside | C_22_H_22_O_10_ |  | R_1_=α-L-Rha, R_2_=OMe, R_3_=H, R_4_=H | (Kavtaradze et al., 2020) |
| 113 | Kaempferol3-*O*-(4″,6″-di-*O*-α-*L*-rhamnopy ranosyl)-*β*-*D*-glucopyranoside | C_33_H_40_O_19_ |  | R_1_=H, R_2_=OH, R_3_=O-[α-L-Rha-(1→2) - [α-L-Rha -(1→6)]- β-D-Gal], R_4_=β-D-Glc | (El Dib et al., 2015) |
| 114 | Quercetin 3-*O*-(4″,6″-di-*O*-α-*L*-rhamnopyra nosyl)-β-*D*-glucopyranoside | C_33_H_40_O_20_ |  | R_1_=H, R_2_=H, R_3_=-[(5-O-trans-p-ferfuloyl)- β-D-apiofuranosyl] - (1→2)]- β-D-Glc, R_4_=H | (El Dib et al., 2015) |
| 115 | Quercetin-3-O-α-L-rhamnopyranosyl-(1→2)-［α-L-rhamnopyranosyl-(1→6)］-β-D-galactopyranosyl}-7-O-β-D-glucopyranoside | C_39_H_50_O_25_ |  | R_1_=H, R_2_=H, R_3_= O-α-L-Rha-(1→2)- β-D-Glc, R_4_=(6-O-trans-p-ferfuloyl)- β-D  -Glc | (Krasteva et al., 2015) |
| 116 | 7-methoxy kaempferol-3-O-α-L-arabinosyl-(1→6)-β-D-galactopyranoside | C_27_H_30_O_15_ |  | R_1_=H, R_2_=Me, R_3_=O-[E]-coumaroyl-3,6-di-O-α-L-fuco-pyranosyl)- β-D-Gal, R_4_=H | (Janibekov et al., 2018) |
| 117 | Kaempferol-3-O-α-L-rhamnopyranosyl-7-O-α-L-rhamnopyr-anosyl-(1→6)-β-D-galactopyranoside | C_33_H_40_O_19_ |  | R_1_=H, R_2_=H, R_3_=O-(4″-6″-di-O-α-L-Rha)- β-D-Glc, R_4_=H | (Janibekov et al., 2018) |
| 118 | Isorhamnetin-3-O-(4-O-[E]-coumaroyl-3,6-α-L-O-fucopyranosyl)-β-D-galactopyranoside | C_43_H_48_O_22_ |  | R_1_=H, R_2_=OH, R_3_=O-(4″-6″-di-O-α-L-Rha)- β-D-Glc, R_4_=H | (Zhou et al., 2021) |
| 119 | Isorhamnetin-3-O-{β-D-apiofuranosyl-(1→2)-［α-L-rhamnopyranosyl-(1→6)］-β-D-galactopyranosyl}-7-O-β-D-glucopyranoside | C_39_H_50_O_25_ |  | R_1_=H, R_2_=OMe, R_3_=O-[β-L-api-(1→2)- α-L-Rha-(1→6)]- β-D-Gal], R_4_=β-D-Glc | (Vasilev et al., 2019) |
| 120 | Isorhamnetin-3-O-[β-D-glucopyranosyl-(1→5)-β-D-apiofuranosyl] - (1→2)-robinobioside | C_39_H_50_O_25_ |  | R_1_=H, R_2_=OMe, R_3_=O-[β-D-Glc(1→5)-β-D-api-[α-L-Rha(1→6)]-β-D-Gal], R_4_=H | (Vasilev et al., 2019) |
| 121 | Isorhamnetin-3-O-(2,6-di-O-α-rhamnopyranosyl-β-D-galactopyranoside)-7-O-β-D-glucopyranoside | C_40_H_52_O_25_ |  | R_1_=H, R_2_=OMe, R_3_=O-(2,6-di-O-α-Rha-β-D-Gal), R_4_=β-D-Glc | (Vasilev et al., 2019) |
| 122 | Isorhamnetin-3-O-robinobioside-7-O-glucoside | C_34_H_42_O_21_ |  | R_1_=H, R_2_=OMe, R_3_=β-D-Gal, R_4_=β-D-Glc | (Vasilev et al., 2019) |
| 123 | Isorhamnetin-3-O-(5′′′-p-hydroxybenzoyl)-β-apiofuranosyl-(1→2) [α-rhamnopyranosyl-(1→6)]-β-galactopyranoside | C_40_H_44_O_22_ |  | R_1_=H, R_2_=OMe, R_3_=O-(5″-p-hydroxybenzoyl)-β-D-api-(1→2) - [ α-L-Rha-(1→6)]- β-D-Gal, R_4_=H | (Zhou et al., 2021) |
| 124 | N-{8-methylquercetin-3-O-［α-L-rhamnopyranosyl-(1→2)-［α-L-rhamnopyranosyl-(1→6)］-β-D-galactopyranosyl］}-3-hydroxypiperidin-2-one | C_39_H_49_NO_22_ |  | R_1_=OH, R_2_=α-L-Rha-(1→2)-[a-L-Rha-(1→6)]- β-D-Gal | (Krasteva et al., 2015) |
| 125 | N-{8-methylkaempferol-3-O-［α-L-rhamnopyranosyl-(1→2)-［α-L-rhamnopyranosyl-(1→6)］-β-D-galactopyranosyl］}-3-hydroxypiperidin-2-one | C_39_H_49_NO_21_ |  | R_1_=H, R_2_=α-L-Rha-(1→2)-[a-L-Rha-(1→6)]- β-D-Gal | (Krasteva et al., 2015) |
| 126 | Astraflavonoid C | C_23_H_28_O_11_ |  |  | (Zhou et al., 2021) |
| 127 | Astragaisoflavan B | C_17_H_18_O_6_ |  |  | (Guo et al., 2016) |
| 128 | (2R,3S)-7,4′-dimethoxy-2′-hydroxyflavanol | C_17_H_18_O_5_ |  |  | (Zhou et al., 2021) |
| 129 | Dolichochaetein A | C_22_H_26_O_6_ |  |  | (W. et al., 2021) |
| 130 | Dolichochaetein B | C_22_H_26_O_5_ |  |  | (W. et al., 2021) |
| 131 | Astragaisoflavan A | C_17_H_18_O_6_ |  |  | (Guo et al., 2016) |
| 132 | Astragaisoflavan C | C_18_H_24_O_7_ |  |  | (Guo et al., 2016) |
| 133 | Astragaisoflavan D | C_34_H_34_O_10_ |  |  | (Guo et al., 2016) |
| 134 | (Z)-2′,5′-dihydroxy-6-methoxyaurone | C_16_H_12_O_5_ |  |  | (Xiao et al., 2014) |
| 135 | 2,2′,5′-trihydroxy-4-methoxychalcone | C_16_H_14_O_5_ |  |  | (Xiao et al., 2014) |
| 136 | (βR)-2,2′,5′,β-tetrahydroxy-4-methoxydihydrochalcone | C_16_H_16_O_6_ |  |  | (Xiao et al., 2014) |
| 137 | (3R,4R)-4,7-hydroxy-2′,3′-dimethoxyisoflavane-4′-O-β-D-glucoside | C_23_H_28_O_11_ |  |  | (Zhou et al., 2021) |
