# Supplementary material for: A review of the botany, phytochemistry, traditional uses, pharmacology, toxicology, and quality control of the Astragalus memeranaceus
Source: Front Pharmacol. 2023 Aug 23;14:1242318. doi: 10.3389/fphar.2023.1242318 (PMC10482111; doi:10.3389/fphar.2023.1242318)
Supplement: Supplementary file 2 [file Table2.docx]

**TABLE 2 Saponins isolated from Huangqi.**

| **No.** | **Name** | [**Molecular**](https://cn.bing.com/dict/search?q=molecular&FORM=BDVSP6&cc=cn)[**formula**](https://cn.bing.com/dict/search?q=formula&FORM=BDVSP6&cc=cn) | **Parent nucleus** | **Substituent** | **Ref** |
| --- | --- | --- | --- | --- | --- |
| 138 | Huangqiyegenin Ⅵ | C_30_H_48_O_5_ |  | R_1_=CH_3_CH(CH_2_)_2_CH(OH)C(CH_3_)_2_OH, R_2_=OH, R_3_=O, R_4_=O | (Zhou et al., 2021) |
| 139 | 20,24-non-epoxy-astragaloside Ⅲ | C_41_H_70_O_15_ |  | R_1_=CH_3_C(OH)CH_2_CH_2_CH(OH)C(CH_3_)_2_OH, R_2_=OH, R_3_=OH, R_4_=O-β-D-Glc-(1→2)-β-D- Xyl | (W. et al., 2021) |
| 140 | 20,24-non-epoxy-astragaloside Ⅳ | C_41_H_70_O_15_ |  | R_1_=CH_3_C(OH)CH_2_CH_2_CH(OH)C(CH_3_)_2_OH, R_2_=OH, R_3_=O-β-D-Glc, R_4_=O-β-D-Xyl | (W. et al., 2021) |
| 141 | 3-O-β-D-xylopyranosyl-6,16-di-O-β-D-glucopyranosyl-3β,6α,16β,24(S)-25-pentahydroxycycloartane | C_47_H_80_O_19_ |  | R_1_=CH_3_CHCH_2_CH_2_CH(OH)C(CH_3_)_2_OH, R_2_=O-β-D-Glc, R_3_=O-β-D-Glc, R_4_=O-β-D-Xyl | (Zhou et al., 2021) |
| 142 | 3-O-β-D-xylopyranosyl-6,16-di-O-β-D-glucopyranosyl-3β,6α,16β,24(S)-25-pentahydroxycycloartane dodecaacetate | C_73_H_106_O_32_ |  | R_1_=CH_3_CHCH_2_CH_2_CH(OH)C(CH_3_)_2_OH, R_2_= O-β-D-Glc, R_3_=O-β-D-Glc, R_4_=α-L-Ara-(1→2)-β-D- Xyl | (Un et al., 2016) |
| 143 | Gomboside A | C_41_H_70_O_14_ |  | R_1_=CH_3_CHCH_2_CH(OH)CH(OH)C(CH_3_)_2_OH, R_2_=OH, R_3_=H, R_4=_O-β-D-Glc-(1→2)-β-D- Xyl | (Maamria et al., 2015) |
| 144 | Gomboside B | C_41_H_68_O_13_ |  | R_1_=CH_3_CHCH_2_CH(OH)CH(OH)C(CH_3_)=CH_2_,R_2_=OH, R_3_=H, R_4_=O-β-D-Glc-(1→2)-β-D- Xyl | (Maamria et al., 2015) |
| 145 | 6α,6β,24(S),25-tetrahydroxycycloartane-3-one | C_30_H_50_O_5_ |  | R_1_=CH_3_CHCH_2_CH_2_CH(OH)C(CH_3_)_2_OH, R_2_=OH, R_3_=OH, R_4_= O | (Aslanipour et al., 2017) |
| 146 | 6-O-β-D-xylopyranosyl-3β,6α,16β,24(S),25-pentahydroxycycloartane | C_35_H_60_O_9_ |  | R_1_=CH_3_CHCH_2_CH_2_CH(OH)C(CH_3_)_2_OH, R_2_=OH, R_3_=O-β-D-Xyl, R_4_=OH | (Aslanipour et al., 2017) |
| 147 | 16β-acetyloxy-3-O-β-D-glucopyranosyloxy-cycloartan-11α,24ξ,25-triol | C_38_H_64_O_11_ |  | R_1_=CH_3_CHCH_2_CH_2_CH(OH)C(CH_3_)_2_OH, R_2_=OAc, R_3_=H, R_4_=O-β-D-Glc | (Zhou et al., 2021) |
| 148 | Gomboside C | C_34_H_54_O_9_ |  | R_1_=β-D-Xyl, R_2_=OAc, R_3_=OMe | (Maamria et al., 2015) |
| 149 | Gomboside D | C_37_H_60_O_9_ |  | R_1_=β-D-Xyl, R_2_=OAc, R_3_=OCH_2_CH_2_CH_2_CH_3_ | (Maamria et al., 2015) |
| 150 | Gomboside E | C_40_H_64_O_14_ |  | R_1_=β-D-Glc-(1→2)-β-D- Xyl, R_2_=OAc, R_3_=OMe | (Maamria et al., 2015) |
| 151 | Gomboside F | C_38_H_62_O_12_ |  | R_1_=β-D-Glc-(1→2)-β-D-Xyl, R_2_=H, R_3_=OM | (Maamria et al., 2015) |
| 152 | 17(R),20(R)-3β,6α,16β-trihydroxycycloar-tanyl-23-carboxylic acid16-lact-one-3-O-β-D-glucopyranoside | C_32_H_50_O_9_ |  | R_1_=β-D-Glc, R_2_=OH, R_3_=O | (Zhou et al., 2021) |
| 153 | 29-O-α-L-rhamnopyranosyl-abrisapogenol B | C_36_H_60_O_8_ |  | R_1_=H, R_2_=β-OH, R_3_=Me, R_4_=CH_2_O-α- L-Rha, R_5_=Me | (Un et al., 2016) |
| 154 | Astraolesaponin A | C_48_H_76_O_18_ |  | R_1_=α-L-Rha-(1→2)-β-D-Glc-(1→2)-β-D-ghucuronopyranosy1, R_2_=O, R_3_=Me, R_4_=Me, R_5_=Me | (Zhang et al., 2016) |
| 155 | Astraolesaponin B | C_48_H_78_O_19_ |  | R_1_=α-L-Rha-(1→2)- β-D-Glc-(1→2)-β-D-ghucuronopyranosy1, R_2_=β-OH, R_3_=Me, R_4_=Me, R_5_=CH_2_OH | (Zhang et al., 2016) |
| 156 | Astraolesaponin E_1_ | C_42_H_64_O_17_ |  | R_1_=β-D-Glc-(1→2)-β-D-ghucuronopyranosy1, R_2_=O, R_3_=CH_2_OH, R_4_=COOH, R_5_=Me | (Zhang et al., 2016) |
| 157 | Astraolesaponin E_2_ | C_41_H_62_O_16_ |  | R_1_=β-D-Xyl-(1→2)-β-D-ghucuronopyranosy1, R_2_=O, R_3_=CH_2_OH, R_4_=COOH, R_5_=Me | (Zhang et al., 2016) |
| 158 | Astraolesaponin D | C_48_H_74_O_19_ |  | R_1_=α-L-Rha-(1→2)-β-D-Glc-(1→2)-β-D-ghucuronopyranosy1 | (Zhang et al., 2016) |
| 159 | Astragaloside III | C_41_H_68_O_14_ |  | R=Glc-(1→2)-Xyl | (Su et al., 2021) |
| 160 | Astragaloside IV | C_41_H_68_O_14_ |  |  | (Su et al., 2021) |
| 161 | Huangqiyenin A | C_36_H_58_O_10_ |  |  | (Su et al., 2021) |
| 162 | Brachyoside B | C_36_H_60_O_10_ |  |  | (Su et al., 2021) |
| 163 | Huangqiyegenin I | C_30_H_48_O_5_ |  |  | (Su et al., 2021) |
| 164 | Huangqiyegenin V | C_30_H_46_O_6_ |  |  | (Su et al., 2021) |
| 165 | Isoastragaloside IV | C_41_H_68_O_14_ |  |  | (Su et al., 2021) |
| 166 | Astragaloside VII | C_47_H_78_O_19_ |  |  | (Su et al., 2021) |
| 167 | Astragaloside II | C_43_H_70_O_15_ |  | R=2-O-Ac-Xyl | (Su et al., 2021) |
| 168 | Isoastragaloside I | C_45_H_72_O_16_ |  | R=2,4-O-Ac_2_-Xyl | (Su et al., 2021) |
| 169 | Acetylastragaloside I | C_47_H_74_O_17_ |  | R=2,3,4-O-Ac_3_-Xyl | (Su et al., 2021) |
| 170 | Isoastragaloside II | C_43_H_70_O_15_ |  | R=3-O-Ac-Xyl | (Su et al., 2021) |
| 171 | Astragaloside I | C_45_H_72_O_16_ |  | R=2,3-O-Ac_2_-Xyl | (Su et al., 2021) |
| 172 | Astramembranoside A | C_42_H_70_O_15_ |  | R=H | (Su et al., 2021) |
| 173 | Agroastragaloside IV | C_49_H_80_O_20_ |  | R=2-O-Ac-Xyl | (Su et al., 2021) |
| 174 | Agroastragaloside III | C_51_H_82_O_21_ |  | R=2,3-O-Ac_2_-Xyl | (Su et al., 2021) |
| 175 | Astragaloside VI | C_47_H_78_O_19_ |  | R=Glc-(1→2)-Xyl, R_1_=Glc, R_2_=H | (Su et al., 2021) |
| 176 | Astragaloside V | C_47_H_78_O_19_ |  | R=Glc-(1→2)-Xyl, R_1_=H, R_2_=H | (Su et al., 2021) |
| 177 | Cycloaraloside A | C_36_H_60_O_10_ |  | R=Glc | (Su et al., 2021) |
| 178 | Astramembrannin II | C_35_H_58_O_9_ |  | R=Xyl | (He and Wang, 1990; Wang et al., 2014a) |
| 179 | Astraverrucin II | C_38_H_62_O_11_ |  | R_1_=2-O-Ac-Glc, R_2_=R_3_=H | (Su et al., 2021) |
| 180 | Astrolanosaponin A_1_ | C_42_H_70_O_15_ |  | R_1_= R_2_= Glc, R_3_=H | (Bian et al., 2006) |
| 181 | Cycloaraloside E | C_42_H_70_O_15_ |  | R_1_= R_3_= Glc, R_2_=H | (Su et al., 2021) |
| 182 | Astrolanosaponin A_2_ | C_44_H_72_O_16_ |  | R_1_=2-O-Ac-Glc, R_2_=H, R_3_= Glc | (Su et al., 2021) |
| 183 | Cyclocanthoside A | C_35_H_60_O_9_ |  | R_1_=Xyl, R_2_=H | (Su et al., 2021) |
| 184 | Astramembranoside B | C_41_H_70_O_14_ |  | R_1_=Glc-(1→2)-Xyl, R_2_=H | (Wang et al., 2015) |
| 185 | Cyclocanthoside E | C_41_H_70_O_14_ |  | R_1_=Xyl, R_2_=Glc | (Su et al., 2021) |
| 186 | Agroastragaloside II | C_43_H_72_O_15_ |  | R_1_=2-O-Ac-Xyl, R_2_=Glc | (Su et al., 2021) |
| 187 | Agroastragaloside I | C_45_H_74_O_16_ |  | R_1_=2,3-O-AC_2_-Xyl, R_2_=Glc | (Su et al., 2021) |
| 188 | Huangqiyenin K | C_37_H_60_O_10_ |  |  | (Su et al., 2021) |
| 189 | Astrolanosaponin B | C_42_H_68_O_15_ |  |  | (Su et al., 2021) |
| 190 | Astrolanosaponin E | C_36_H_60_O_11_ |  |  | (Wen et al., 2010) |
| 191 | Astrolanosaponin D | C_36_H_60_O_10_ |  |  | (Su et al., 2021) |
| 192 | Huangqiyenin B | C_36_H_60_O_10_ |  |  | (Su et al., 2021) |
| 193 | Mongholicoside II | C_38_H_62_O_11_ |  |  | (Wang et al., 2014a) |
| 194 | Huangqiyenin F | C_40_H_64_O_12_ |  | R= H | (Su et al., 2021) |
| 195 | Huangqiyenin G | C_40_H_62_O_13_ |  | R=OH | (Su et al., 2021) |
| 196 | Huangqiyenin E | C_42_H_66_O_14_ |  | R= β O-Ac | (Su et al., 2021) |
| 197 | Huangqiyenin H | C_40_H_60_O_13_ |  |  | (Su et al., 2021) |
| 198 | Huangqiyenin J | C_40_H_62_O_12_ |  |  | (Su et al., 2021) |
| 199 | Huangqiyenin I | C_40_H_64_O_13_ |  |  | (Su et al., 2021) |
| 200 | Azukisaponin V | C_48_H_78_O_18_ |  | R= Rha- (1→2) - Glc-(1→2)- GlcA | (Su et al., 2021) |
| 201 | Astragaloside VIII | C_47_H_76_O_17_ |  | R=Rha-(1→2)-Xyl-(1→2)-GlcA | (Su et al., 2021) |
| 202 | Soyasaponin I | C_48_H_78_O_18_ |  | R=Rha-(1→2)-Gal-(1→2)-GlcA | (Su et al., 2021) |
| 203 | Mongholicoside I | C_36_H_60_O_9_ |  |  | (Su et al., 2021) |
| 204 | Huangqiyenin L | C_43_H_70_O_14_ |  |  | (Su et al., 2021) |
| 205 | Aleksandroside I | C_36_H_62_O_10_ |  |  | (Su et al., 2021) |
| 206 | Alexandroside I | C_36_H_62_O_10_ |  |  | (Su et al., 2021) |
| 207 | Mongholicoside A | C_36_H_62_O_11_ |  | R=β-OH | (Su et al., 2021) |
| 208 | Mongholicoside B | C_36_H_60_O_11_ |  | R= OH | (Su et al., 2021) |
| 209 | Soyasapogenol B | C_30_H_50_O_3_ |  | R= β OH | (Su et al., 2021) |
| 210 | Soyasapogenol E | C_30_H_48_O_3_ |  | R= =O | (Su et al., 2021) |
| 211 | (3β,22β)‐olean‐12‐ene‐  3,22,24,29‐tetrol | C_30_H_50_O_4_ |  |  | (Su et al., 2021) |
| 212 | (3β,21α)‐olean‐12‐ene‐  3,21,24‐triol | C_30_H_50_O_3_ |  |  | (Hao et al., 2016) |
| 213 | Astroolesaponin A | C_48_H_76_O_18_ |  | R_1_=Rha-(1→2)-Glc-(1→2)-GlcA, R_2_=H | (Su et al., 2021) |
| 214 | Cloversaponin III | C_42_H_64_O_16_ |  | R_1_=Glc-(1→2)-GlcA, R_2_=H | (Su et al., 2021) |
| 215 | Astroolesaponin E_1_ | C_42_H_64_O_17_ |  | R_1_=Glc-(1→2)-GlcA, R_2_=OH | (Su et al., 2021) |
| 216 | Astroolesaponin E_2_ | C_41_H_62_O_16_ |  | R_1_=Xyl-(1→2)-GlcA, R_2_=OH | (Su et al., 2021) |
| 217 | Astraisoolesaponin B | C_42_H_62_O_16_ |  | R_1_=Glc-(1→2)-GlcA, R_2_=H | (Su et al., 2021) |
| 218 | Astraisoolesaponin A_2_ | C_42_H_62_O_17_ |  | R_1_=Glc-(1→2)-GlcA, R_2_=OH | (Su et al., 2021) |
| 219 | Astraisoolesaponin A_3_ | C_41_H_60_O_16_ |  | R_1_=Xyl-(1→2)-GlcA, R_2_=OH | (Su et al., 2021) |
| 220 | Astraisoolesaponin A_1_ | C_48_H_72_O_21_ |  | R_1_=Rha-(1→2)-Glc-(1→2)-GlcA, R_2_=OH | (Su et al., 2021) |
| 221 | Astroolesaponin F | C_49_H_78_O_18_ |  | R=Rha-(1→2)-Glc-(1→2)-GlcA methyl ester | (Su et al., 2021) |
| 222 | Azukisaponin V | C_49_H_80_O_18_ |  | R=Rha-(1→2)-Glc-(1→2)-GlcA | (Su et al., 2021) |
| 223 | Astragaloside VIII | C_48_H_78_O_17_ |  | R=Rha-(1→2)-Xyl-(1→2)-GlcA | (Su et al., 2021) |
| 224 | Robinioside F | C_48_H_78_O_19_ |  |  | (Su et al., 2021) |
| 225 | Astroolesaponin C_1_ | C_43_H_68_O_16_ |  | R=Glc-(1→2)-GlcA methyl ester | (Su et al., 2021) |
| 226 | Robinioside B | C_48_H_76_O_20_ |  | R=Rha-(1→2)-Glc-(1→2)-GlcA | (Su et al., 2021) |
| 227 | Astroolesaponin C_2_ | C_49_H_78_O_20_ |  | R=Rha-(1→2)-Glc-(1→2)-GlcA methyl ester | (Su et al., 2021) |
| 228 | Astroolesaponin D | C_48_H_74_O_19_ |  | R=Rha-(1→2)-Glc-(1→2)-GlcA | (Su et al., 2021) |
| 229 | Astroolesaponin B | C_48_H_78_O_19_ |  | R=Rha-(1→2)-Glc-(1→2)-GlcA | (Su et al., 2021) |
| 230 | Lupeol | C_30_H_50_O |  |  | (Su et al., 2021) |
| 231 | Ursolic acid | C_30_H_48_O_3_ |  |  | (Su et al., 2021)a |
